# Analysis of Critical Thinking Path of College Students Under STEAM Course

**DOI:** 10.3389/fpsyg.2021.723185

**Published:** 2021-09-03

**Authors:** Jingrong Sha, Hong Shu, Zhaocao Kan

**Affiliations:** ^1^College of Educational Science and Technology, Northwest Minzu University, Lanzhou, China; ^2^College of Teacher Education, East China Normal University, Shanghai, China

**Keywords:** STEAM, STEAM course, critical thinking, academic engagement, science skills

## Abstract

This study examined the differences in critical thinking levels among students with different levels of academic engagement in STEAM courses. In this study, 30 college students were selected as subjects. Before experimenting, they received the academic engagement test and were divided into high, medium, and low groups based on their performance. Then, each group received three STEAM sessions and was asked to complete a topic discussion task. The results show that there are significant differences in the critical thinking level of students with different levels of academic engagement. Specifically, the students with a medium level of academic engagement had the highest critical thinking. Research has shown that the level of academic engagement affects the critical thinking of students in STEAM courses.

## Introduction

Critical thinking is one of the twenty-first skills centuries for contemporary college students, ranking as the most sought-after higher-order thinking skills, along with creativity, collaboration, and problem-solving (Lai and Viering, 2012; Vasilyev et al., 2015; Podolsky and Pogozhina, 2017). Critical thinking is defined as purposeful, self-calibrated judgment. This kind of judgment manifests itself in interpretation, analysis, evaluation, inference, and the explanation of the evidence, concept, method, standard, or context on which the judgment depends (Nair and Lynnette Leeseberg, 2013).

The studies indicated that the STEAM course has the potential to improve the critical thinking of students (Allamin et al., 2018; Siregar et al., 2019). The STEAM course is an interdisciplinary and integrated course that combines science, technology, engineering, art, and mathematics (Yakman, 2008; Corbo et al., 2014; Hwang, 2017). In the STEAM course, students can focus on the specific problems rather than being confined to a single subject boundary, and they can practice their thinking from different perspectives and develop cross-border communication in the context of diversified development (Yakman, 2008; Corbo et al., 2014; Hwang, 2017; Hatlevik, 2018). The STEAM course is designed to guide learners to develop problem-solving, critical thinking, and collaboration skills (Tillinghast et al., 2015).

The previous studies results are not consistent on whether STEAM course improves the critical thinking of students. For example, Ridwan et al. (2020) implemented a STEAM course based on a smoke absorber project in a high school chemistry course. Through simple analysis of online discussion texts, the study found that STEAM courses can promote the development of the critical thinking of students (Ridwan et al., 2020). However, other researchers have found a different result. For example, Ho Sha (2019) explored the changes in the critical thinking level of students before and after the implementation of the STEAM course and found that the critical thinking level of students did not improve significantly.

The cultivation of critical thinking in STEAM courses may be influenced by academic engagement. In the study, each student has a different degree of academic engagement (Curry, 1984; Nystrand, 1989; Cancelli, 1993). The research found that academic investment can affect the self-efficacy of students (Yüksel and Alci, 2012; Samareh and Kezri, 2016). Specifically, students with high education have more self-efficacy (Samareh and Kezri, 2016). In addition, the study found that self-efficacy and critical thinking were significantly correlated, specifically, the higher the self-efficacy of students, the higher the level of academic engagement (McKinnon, 2012; Dong, 2016). Then, academic engagement may affect critical thinking in the STEAM curriculum.

This study aimed to examine whether the implementation of STEAM courses had significant differences in the development of critical thinking levels of students at different levels of academic engagement (high, medium, and low). Based on previous research, this study hypothesizes that academic engagement affects the critical thinking of students in STEAM courses. Specifically, the higher the level of academic engagement, the higher is the critical thinking level of students in STEAM courses.

## Methods

### Participants

The participants in this study were 30 college students (14 male students and 16 female students). The average age of the participants was 20.43 (SD = 0.89). All participants received a small gift worth 30 RMB after the completion of the experiment. The study protocol was approved by the local Academic Committee.

This experiment was an in-subject experiment design, and all subjects received three STEAM courses. The subjects were asked to fill out the questionnaire before receiving the experimental treatment, and according to the results, the subjects were divided into groups with high, medium, and low academic involvement.

### Measures

#### Academic Engagement Scale

The Academic Engagement Scale (Awang-Hashim and Sani, 2008) is used to measure the academic engagement of students in school learning activities. The scale is divided into three dimensions: “behavioral engagement,” “cognitive engagement,” and “emotional engagement.” The scale has a total of 29 items, which consist of eight items of behavioral engagement, 10 items of cognitive engagement, and 11 items of affective engagement. The scale uses Likert-five assessment, with 1 representing “completely inconsistent” and 5 representing “completely consistent.” The higher the score, the higher is the degree of academic engagement. The internal consistency coefficient of the scale was 0.851, among which the behavioral engagement dimension coefficient was 0.782, the cognitive engagement dimension coefficient was 0.835, and the affective engagement dimension coefficient was measured to 0.742.

#### Critical Thinking Coding Tool

The study adopted the critical thinking coding tool proposed by Murphy (2004) and added the “Null” dimension (encoding topics unrelated to the course and opinions purely expressing emotions) in this study. The critical thinking coding tool is divided into six dimensions: recognition, understanding, analysis, evaluation, creation, and null value, including a total of 25 specific indicators.

#### Online Collaboration Platform

Each group establishes a group online cooperative learning community through QQ. It is a widely used instant messaging software in China, which can realize voice and text communication, file transfer, and other functions. Before the experiment, the participants were informed that the teacher would post the course tasks that needed to be solved in each class on the QQ group and asked them to discuss according to the task requirements proposed by the teacher, and finally put forward a group plan.

### Procedure

Before the formal experiment, each participant was required to complete the Academic Engagement Scale. Then, the experimenter divided the participants into three groups according to the academic engagement questionnaire: high, medium, and low. After that, all participants were given three STEAM sessions. Each course lasts 90 min, and the contents of the three courses are shown in Table 1.

**Table 1:** Steam course design and implementation plan.

**STEAM course**	**Engineering design process**	**STEAM event plan**	**Activity content**	**Discussion theme**	**STEAM element integration**
1	1. Identify the Theme/requirements	– Students identify subject-related needs	– Searching and summarizing the content of engineering thinking, ADDIE teaching thought, structure, design and so on online in groups	Combined with ADDIE's instructional design analysis and engineering ideas, this paper discusses how to do a good job in online course design	Connotation, structure, and design of engineering thinking and overall course process design—integration of science content and engineering content
	2. Gather ideas/explore information	– Students use technology to find solutions – The students did experiments related to the STEAM event			
2	3. Solution design	– Students put their heads together to come up with more possible solutions – Students design online course plans	– Searching online course design examples by myself in groups – Collect the similarities and differences of cases through group discussion – Plan the overall curriculum by myself in combination with scientific content and engineering thinking	Discuss the design of the online learning program based on the results of the demand survey and analysis	Mathematical statistics for needs analysis, learning activity design—the integration of mathematical content and engineering content
	4. Implementation and development	– Students take online courses	– Design the needs analysis questionnaire – Distribution and collation of data results – Based on the analysis results of learning rules and characteristics, online learning activities were designed in the form of group discussion		
3	5. Test, evaluate, and design improvements	– Students test and evaluate online courses – Students adapt their online courses to their needs	– Technical support and interactive interface design (graphic design, interaction design, user research, etc.) for the group's online course – Online course evaluation test was conducted for each group and improved according to the actual situation	Combined with the content of online platform technical support and interface design, how to do a good job of online learning support and service?	Online teaching and learning support, friendly interface design—the integration of art and technology content
	6. Display of works	– Students share their designs and successes with the class through demonstrations	– Students report online courses in groups, highlight the advantages and innovations, and evaluate them by their fellow teachers		

At the end of the three sessions, the online discussion texts were collated for coding and lag sequence analysis (LSA). First, the two researchers coded the code together. They are familiar with coding tools for critical thinking and have discussions before coding. In the coding process, the two researchers separately encoded the text of the online discussion. After the formal coding was completed, 154 texts were randomly checked by the two coders to verify the consistency of the coding results of the two researchers. The results show that the Kappa consistency coefficient is 0.75, which indicates that the data encoding has good reliability and can be used for LSA. Second, GSEQ software is used to analyze the lag sequence. Lagging sequence analysis produces two important tables, namely, behavior conversion frequency table and adjustment residual table. According to the theory of LSA, the *Z*-score >1.96 indicates that this behavior path is significant (Sjalander et al., 1996). Finally, the behavior transformation diagram is drawn according to the important behavior sequence. The result is research on critical thinking levels.

## Results

### The Critical Thinking Pathways of Participants With Different Levels of Academic Engagement After the First STEAM Course

According to the coding results, the characteristics of critical thinking pathways of the low, medium, and high academic engagement group students after the first course were analyzed. The specific results are analyzed as follows.

The results showed that, for students with low academic engagement, there are four kinds and five effective single sequences in this stage, such as R2→U5, U5→A4, R1→E1, and C1→C2, as shown in Table 2. For the first time to talk about the single sequence including identification agree with to understand answer questions (R2→U5), understanding to answer the question—analysis explanation view (U52→A4), preliminary identification problem to determine the effectiveness of the current information and value point (R12→E1), project implementation plan to create design new ideas (C12→C2) sequence, respectively 1, 2, 1, 1.

**Table 2:** Behavior switching frequency of critical thinking process in the low engagement group.

**Given:**	**R1**	**R2**	**U1**	**U2**	**U3**	**U4**	**U5**	**U6**	**A1**	**A2**	**A3**	**A4**	**A5**	**A6**	**E1**	**E2**	**E3**	**E4**	**E5**	**E6**	**C1**	**C2**	**C3**	**N1**	**N2**	**Totals**
R1	0	0	0	0	0	0	0	0	0	0	0	0	0	0	0	1	0	0	0	0	0	0	0	0	0	1
R2	0	0	0	0	0	0	1	0	0	0	0	0	0	0	0	0	0	0	0	0	0	0	0	0	0	1
U1	0	0	0	0	0	0	0	0	0	0	0	0	0	0	0	0	0	0	0	0	0	0	0	0	0	0
U2	0	0	0	0	0	0	0	0	0	0	0	0	0	0	0	0	0	0	0	0	0	0	0	0	0	0
U3	0	0	0	0	0	0	0	0	0	0	0	0	0	0	0	0	0	0	0	0	0	0	0	0	0	0
U4	0	0	0	0	0	0	0	0	0	0	0	0	0	0	0	0	0	0	0	0	0	0	0	0	0	0
U5	0	0	0	0	0	0	0	0	0	0	0	2	0	0	0	0	0	0	0	0	0	0	0	0	0	2
U6	0	0	0	0	0	0	0	0	0	0	0	0	0	0	0	0	0	0	0	0	0	0	0	0	0	0
A1	0	0	0	0	0	0	0	0	0	0	0	0	0	0	0	0	0	0	0	0	0	0	0	0	0	0
A2	0	0	0	0	0	0	0	0	0	0	0	0	0	0	0	0	0	0	0	0	0	0	0	0	0	0
A3	0	0	0	0	0	0	0	0	0	0	0	0	0	0	0	0	0	0	0	0	0	0	0	0	0	0
A4	0	0	0	0	0	0	0	0	0	0	0	0	0	0	0	0	0	0	0	0	0	0	0	0	0	0
A5	0	0	0	0	0	0	0	0	0	0	0	0	0	0	0	0	0	0	0	0	0	0	0	0	0	0
A6	0	0	0	0	0	0	0	0	0	0	0	0	0	0	0	0	0	0	0	0	0	0	0	0	0	0
E1	0	0	0	0	0	0	0	0	0	0	0	0	0	0	0	0	0	0	0	0	0	0	0	0	0	0
E2	0	0	0	0	0	0	0	0	0	0	0	0	0	0	0	0	0	0	0	0	0	0	0	0	0	0
E3	0	0	0	0	0	0	0	0	0	0	0	0	0	0	0	0	0	0	0	0	0	0	0	0	0	0
E4	0	0	0	0	0	0	0	0	0	0	0	0	0	0	0	0	0	0	0	0	0	0	0	0	0	0
E5	0	0	0	0	0	0	0	0	0	0	0	0	0	0	0	0	0	0	0	0	0	0	0	0	0	0
E6	0	0	0	0	0	0	0	0	0	0	0	0	0	0	0	0	0	0	0	0	0	0	0	0	0	0
C1	0	0	0	0	0	0	0	0	0	0	0	0	0	0	0	0	0	0	0	0	0	1	0	0	0	1
C2	0	0	0	0	0	0	0	0	0	0	0	0	0	0	0	0	0	0	0	0	0	0	0	0	0	0
C3	0	0	0	0	0	0	0	0	0	0	0	0	0	0	0	0	0	0	0	0	0	0	0	0	0	0
N1	0	0	0	0	0	0	0	0	0	0	0	0	0	0	0	0	0	0	0	0	0	0	0	0	0	0
N2	0	0	0	0	0	0	0	0	0	0	0	0	0	0	0	0	0	0	0	0	0	0	0	0	0	0
Totals	0	0	0	0	0	0	1	0	0	0	0	2	0	0	0	1	0	0	0	0	0	1	0	0	0	5

**Figure 1:**
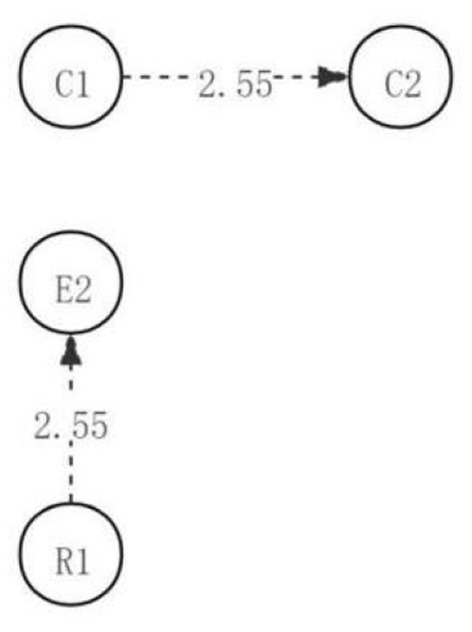
The critical thinking discussion activity path diagram of the low engagement group.

The adjusted residual was calculated by GSEQ software (as shown in Table 3). Among them, there are two significant sequences with residual values >1.96, namely, preliminary identification of problems → judgment of the validity and value points of current information (R1→E1), application of implementation plan → creation of new ideas (C1→C2). In addition, according to the adjusted sequence of significant behavioral residuals, a complete directed path diagram of learning activities is generated, as shown in Figure 1. As can be seen from the visual path diagram discussed in the first discussion, students in the low engagement group initially identified the problem and then judged the validity and value of the information provided. Students will be inspired to put forward new ideas, strategies, and methods based on applying new ideas and plans.

**Table 3:** Behavioral adjustment residual values of critical thinking process in the low engagement group.

**Given:**	**R1**	**R2**	**U1**	**U2**	**U3**	**U4**	**U5**	**U6**	**A1**	**A2**	**A3**	**A4**	**A5**	**A6**	**E1**	**E2**	**E3**	**E4**	**E5**	**E6**	**C1**	**C2**	**C3**	**N1**	**N2**
R1	0	0	0	0	0	0	−0.72	0	0	0	0	−0.83	0	0	0	2.55^*^	0	0	0	0	0	−0.51	0	0	0
R2	0	0	0	0	0	0	1.45	0	0	0	0	−0.83	0	0	0	−0.51	0	0	0	0	0	−0.51	0	0	0
U1	0	0	0	0	0	0	0	0	0	0	0	0	0	0	0	0	0	0	0	0	0	0	0	0	0
U2	0	0	0	0	0	0	0	0	0	0	0	0	0	0	0	0	0	0	0	0	0	0	0	0	0
U3	0	0	0	0	0	0	0	0	0	0	0	0	0	0	0	0	0	0	0	0	0	0	0	0	0
U4	0	0	0	0	0	0	0	0	0	0	0	0	0	0	0	0	0	0	0	0	0	0	0	0	0
U5	0	0	0	0	0	0	0	0	0	0	0	1.67	0	0	0	−1.02	0	0	0	0	0	−1.02	0	0	0
U6	0	0	0	0	0	0	0	0	0	0	0	0	0	0	0	0	0	0	0	0	0	0	0	0	0
A1	0	0	0	0	0	0	0	0	0	0	0	0	0	0	0	0	0	0	0	0	0	0	0	0	0
A2	0	0	0	0	0	0	0	0	0	0	0	0	0	0	0	0	0	0	0	0	0	0	0	0	0
A3	0	0	0	0	0	0	0	0	0	0	0	0	0	0	0	0	0	0	0	0	0	0	0	0	0
A4	0	0	0	0	0	0	0	0	0	0	0	0	0	0	0	0	0	0	0	0	0	0	0	0	0
A5	0	0	0	0	0	0	0	0	0	0	0	0	0	0	0	0	0	0	0	0	0	0	0	0	0
A6	0	0	0	0	0	0	0	0	0	0	0	0	0	0	0	0	0	0	0	0	0	0	0	0	0
E1	0	0	0	0	0	0	0	0	0	0	0	0	0	0	0	0	0	0	0	0	0	0	0	0	0
E2	0	0	0	0	0	0	0	0	0	0	0	0	0	0	0	0	0	0	0	0	0	0	0	0	0
E3	0	0	0	0	0	0	0	0	0	0	0	0	0	0	0	0	0	0	0	0	0	0	0	0	0
E4	0	0	0	0	0	0	0	0	0	0	0	0	0	0	0	0	0	0	0	0	0	0	0	0	0
E5	0	0	0	0	0	0	0	0	0	0	0	0	0	0	0	0	0	0	0	0	0	0	0	0	0
E6	0	0	0	0	0	0	0	0	0	0	0	0	0	0	0	0	0	0	0	0	0	0	0	0	0
C1	0	0	0	0	0	0	−0.72	0	0	0	0	−0.83	0	0	0	−0.51	0	0	0	0	0	2.55^*^	0	0	0
C2	0	0	0	0	0	0	0	0	0	0	0	0	0	0	0	0	0	0	0	0	0	0	0	0	0
C3	0	0	0	0	0	0	0	0	0	0	0	0	0	0	0	0	0	0	0	0	0	0	0	0	0
N1	0	0	0	0	0	0	0	0	0	0	0	0	0	0	0	0	0	0	0	0	0	0	0	0	0
N2	0	0	0	0	0	0	0	0	0	0	0	0	0	0	0	0	0	0	0	0	0	0	0	0	0

* *Indicates that the Z-score value is significant*.

The results showed that for students with medium academic engagement, U5→A4, A4→A5 are generated in this stage, with a total of 25 kinds and 46 effective single sequences, as shown in Table 4. The single sequence generated in the first discussion includes understanding and answering questions → analyzing and explaining viewpoints (U5→A4), analyzing and explaining viewpoints → decomposition of problem viewpoints (A4→A5). There are six and eight sequences, respectively, and there are relatively few process sequences from creating (C).

**Table 4:** Behavioral adjustment residual values of critical thinking process in the medium engagement group.

**Given:**	**R1**	**R2**	**U1**	**U2**	**U3**	**U4**	**U5**	**U6**	**A1**	**A2**	**A3**	**A4**	**A5**	**A6**	**E1**	**E2**	**E3**	**E4**	**E5**	**E6**	**C1**	**C2**	**C3**	**N1**	**N2**	**Totals**
R1	0	1	0	0	0	0	0	0	0	0	0	0	0	0	0	0	0	0	0	0	0	0	0	0	0	1
R2	0	0	0	1	0	0	0	0	1	0	0	3	0	0	0	0	0	0	1	0	0	0	0	0	0	6
U1	0	0	0	0	0	0	0	0	0	0	0	0	0	0	0	0	0	0	0	0	0	0	0	0	0	0
U2	0	0	0	0	0	0	0	0	0	0	0	0	0	0	0	0	0	0	0	0	0	0	0	0	0	0
U3	0	0	0	0	0	0	0	0	0	0	0	0	0	0	0	0	0	0	0	0	0	0	0	0	0	0
U4	0	0	0	0	0	0	0	0	0	0	0	0	0	0	0	0	0	0	0	0	0	0	0	0	0	0
U5	0	0	0	0	0	0	0	0	0	0	0	6	0	0	0	0	0	0	2	0	0	0	0	0	0	8
U6	0	0	0	0	0	0	0	0	0	0	0	0	0	0	0	0	0	0	0	0	0	0	0	0	0	0
A1	0	0	0	0	0	0	0	0	0	0	0	1	1	0	1	0	1	0	0	0	0	0	0	0	0	4
A2	0	0	0	0	0	0	0	0	0	0	1	2	0	0	0	0	0	0	0	0	0	0	0	0	0	3
A3	0	0	0	0	0	0	0	0	0	0	0	1	0	0	0	0	0	0	0	0	0	0	0	0	0	1
A4	0	0	0	0	0	0	0	0	0	0	0	0	8	0	0	0	0	0	3	0	0	0	0	0	0	11
A5	0	0	0	0	0	0	0	0	0	0	0	0	0	0	0	0	0	0	1	0	0	1	0	0	0	2
A6	0	0	0	0	0	0	0	0	0	0	0	0	0	0	0	0	0	0	0	0	0	0	0	0	0	0
E1	0	0	0	0	0	0	0	0	0	0	0	0	0	0	0	1	0	0	0	0	0	0	0	0	0	1
E2	0	0	0	0	0	0	0	0	0	0	0	0	0	0	0	0	1	0	3	0	0	0	0	0	0	4
E3	0	0	0	0	0	0	0	0	0	0	0	0	0	0	0	0	0	0	0	1	0	0	0	0	0	1
E4	0	0	0	0	0	0	0	0	0	0	0	0	0	0	0	0	0	0	0	0	0	0	0	0	0	0
E5	0	0	0	0	0	0	0	0	0	0	0	0	0	0	0	0	0	0	0	2	0	1	0	0	0	3
E6	0	0	0	0	0	0	0	0	0	0	0	0	0	0	0	0	0	0	0	0	0	0	0	0	0	0
C1	0	0	0	0	0	0	0	0	0	0	0	0	0	0	0	0	0	0	0	0	0	1	0	0	0	1
C2	0	0	0	0	0	0	0	0	0	0	0	0	0	0	0	0	0	0	0	0	0	0	0	0	0	0
C3	0	0	0	0	0	0	0	0	0	0	0	0	0	0	0	0	0	0	0	0	0	0	0	0	0	0
N1	0	0	0	0	0	0	0	0	0	0	0	0	0	0	0	0	0	0	0	0	0	0	0	0	0	0
N2	0	0	0	0	0	0	0	0	0	0	0	0	0	0	0	0	0	0	0	0	0	0	0	0	0	0
Totals	0	1	0	1	0	0	0	0	1	0	1	13	9	0	1	1	2	0	10	3	0	3	0	0	0	46

**Figure 2:**
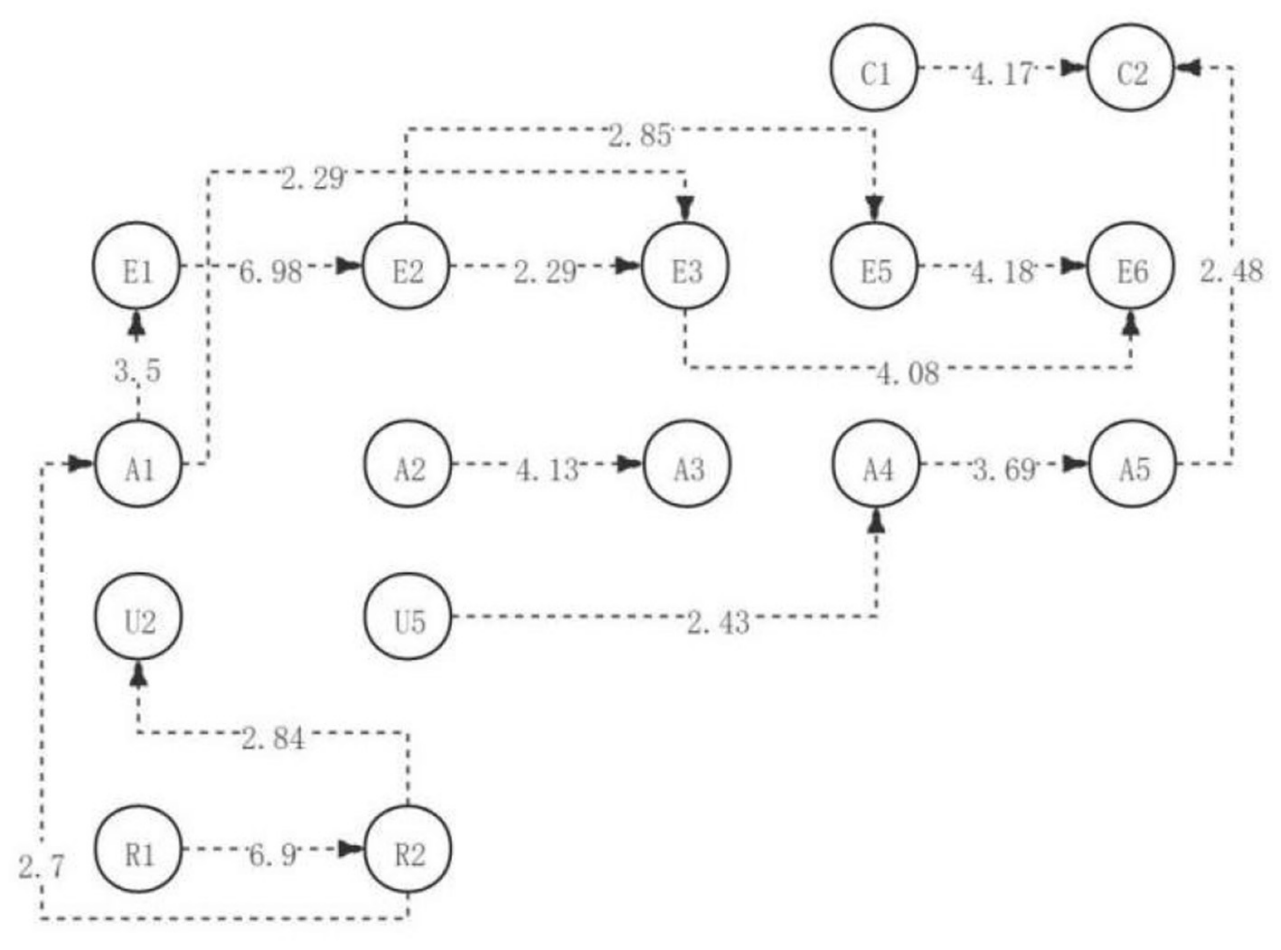
The critical thinking discussion activity path diagram of the medium engagement group.

As can be seen from the visual path diagram of the first discussion, after the students understand and answer the questions, they then analyze and explain the questions, and further decompose the views of the current problems, to create new views for the current problems to be solved (U5→A4→A5→C2). During the discussion, students usually create new ideas (C1→C2) based on creating and implementing new ideas, new conclusions, or new plans. The next step in the classification of evidence is usually to further analyze the similarities and differences between opinions (A2→A3). After the preliminary identification of repeated problems, most students responded to agree with the current specific problems. Then, they will join themselves to solve the current problems of new or new ways of thinking, next, the difference in the behavior way, part of the society, and to detect the current point of view of the consistency and inconsistency. Finally, the students will show their acceptance or refutation of the views and explain why and list the arguments (R1→R2→A1→E3→E6). The other students will first judge the validity of the information sources and the value of the information itself, and then, they further criticize the views themselves, and then put forward their views and find relevant arguments to support the arguments. Finally, we will summarize and state the acceptance or refutation and explain the reasons for the argument (R1→R2→A1→E1→E2→E5→E6). In this process, some students will first test the consistency and inconsistency after criticizing the opinions. Then, directly discuss the acceptance or refutation and explain the reasons for the argument (R1→R2→A1→E1→E2→E3→E6). The common points presented by the visualization path chart are: students will first identify and clarify the existing views, problems, and contradictions, then, explain in-depth, organize the known information, clarify the unknown information, decompose the basic elements of the opinions or problems, and finally clearly criticize and judge the information or opinions. In addition, according to the adjusted sequence of significant behavioral residuals, a complete directed path diagram of learning activities is generated, as shown in Figure 2.

Among them, there were 15 significant sequences with residual values greater than 1.96 (as shown in Table 5).In other words, identify the restatement question → identify the agreement question (R1→R2), identify the agreement opinion → understand and answer the question (R2→U2), identify the agreement question → add new thinking behavior (R2→A1), understand and answer the question → analyze and explain the opinion (U5→A4), add new thinking behavior → judge the validity of information (A1→E1), add new thinking behavior → test 1Causes and inconsistencies (A1→E3), evidence classification → analysis of opinion similarities and differences (A2→A3), analysis and interpretation of opinion → decomposition of problem opinion (A4→A5), decomposition of problem opinion → creation of new opinion (A5→C2), judgment of information validity → critical opinion hypothesis (E1→E2), critical opinion hypothesis → detection of consistency and inconsistencies (E2→E3), critical opinion hypothesis → evidence supporting the argument (E2→E5), detection of consistency and inconsistency → refute acceptance and evidence (E3→E6), evidence supporting the argument → refute acceptance and evidence (E5→E6), evidence supporting the argument → create a new idea (E5→C2), application of implementation plan → create a new idea (C1→C2).

**Table 5:** Behavioral adjustment residual values of critical thinking process in the medium engagement group.

**Given:**	**R1**	**R2**	**U1**	**U2**	**U3**	**U4**	**U5**	**U6**	**A1**	**A2**	**A3**	**A4**	**A5**	**A6**	**E1**	**E2**	**E3**	**E4**	**E5**	**E6**	**C1**	**C2**	**C3**	**N1**	**N2**
R1	0	6.9^*^	0	−0.14	0	0	0	0	−0.15	0	−0.14	−0.71	−0.47	0	−0.14	−0.15	−0.2	0	−0.51	−0.25	0	−0.25	0	0	0
R2	0	0	0	2.84^*^	0	0	0	0	2.7^*^	0	−0.37	0.7	−1.24	0	−0.37	−0.38	−0.53	0	−0.25	−0.65	0	−0.65	0	0	0
U1	0	0	0	0	0	0	0	0	0	0	0	0	0	0	0	0	0	0	0	0	0	0	0	0	0
U2	0	0	0	0	0	0	0	0	0	0	0	0	0	0	0	0	0	0	0	0	0	0	0	0	0
U3	0	0	0	0	0	0	0	0	0	0	0	0	0	0	0	0	0	0	0	0	0	0	0	0	0
U4	0	0	0	0	0	0	0	0	0	0	0	0	0	0	0	0	0	0	0	0	0	0	0	0	0
U5	0	−0.46	0	−0.43	0	0	0	0	−0.45	0	−0.43	2.43^*^	−1.45	0	−0.43	−0.45	−0.62	0	0.39	−0.76	0	−0.76	0	0	0
U6	0	0	0	0	0	0	0	0	0	0	0	0	0	0	0	0	0	0	0	0	0	0	0	0	0
A1	0	−0.31	0	−0.29	0	0	0	0	0	0	−0.29	−0.46	0.39	0	3.5^*^	−0.3	2.29^*^	0	−1.07	−0.52	0	−0.52	0	0	0
A2	0	−0.26	0	−0.25	0	0	0	0	−0.26	0	4.13^*^	1.1	−0.84	0	−0.25	−0.26	−0.36	0	−0.91	−0.44	0	−0.44	0	0	0
A3	0	−0.15	0	−0.14	0	0	0	0	−0.15	0	0	1.26	−0.48	0	−0.14	−0.15	−0.2	0	−0.52	−0.25	0	−0.25	0	0	0
A4	0	−0.69	0	−0.65	0	0	0	0	−0.68	0	−0.66	0	3.69^*^	0	−0.66	−0.68	−0.94	0	−0.3	−1.16	0	−1.16	0	0	0
A5	0	−0.23	0	−0.22	0	0	0	0	−0.23	0	−0.22	−1.12	0	0	−0.22	−0.23	−0.32	0	0.85	−0.39	0	2.48^*^	0	0	0
A6	0	0	0	0	0	0	0	0	0	0	0	0	0	0	0	0	0	0	0	0	0	0	0	0	0
E1	0	−0.15	0	−0.14	0	0	0	0	−0.15	0	−0.14	−0.72	−0.48	0	0	6.98^*^	−0.2	0	−0.52	−0.25	0	−0.25	0	0	0
E2	0	−0.31	0	−0.29	0	0	0	0	−0.3	0	−0.29	−1.49	−0.99	0	−0.29	0	2.29^*^	0	2.85^*^	−0.52	0	−0.52	0	0	0
E3	0	−0.15	0	−0.14	0	0	0	0	−0.15	0	−0.14	−0.73	−0.48	0	−0.14	−0.15	0	0	−0.52	4.08^*^	0	−0.25	0	0	0
E4	0	0	0	0	0	0	0	0	0	0	0	0	0	0	0	0	0	0	0	0	0	0	0	0	0
E5	0	−0.29	0	−0.28	0	0	0	0	−0.29	0	−0.28	−1.41	−0.94	0	−0.28	−0.29	−0.4	0	0	4.18^*^	0	1.84	0	0	0
E6	0	0	0	0	0	0	0	0	0	0	0	0	0	0	0	0	0	0	0	0	0	0	0	0	0
C1	0	−0.15	0	−0.14	0	0	0	0	−0.15	0	−0.14	−0.71	−0.47	0	−0.14	−0.15	−0.2	0	−0.51	−0.25	0	4.17^*^	0	0	0
C2	0	0	0	0	0	0	0	0	0	0	0	0	0	0	0	0	0	0	0	0	0	0	0	0	0
C3	0	0	0	0	0	0	0	0	0	0	0	0	0	0	0	0	0	0	0	0	0	0	0	0	0
N1	0	0	0	0	0	0	0	0	0	0	0	0	0	0	0	0	0	0	0	0	0	0	0	0	0
N2	0	0	0	0	0	0	0	0	0	0	0	0	0	0	0	0	0	0	0	0	0	0	0	0	0

* *Indicates that the Z–score value is significant*.

The results showed that for students with high academic engagement, this stage mainly produces 18 kinds of R2→A4 and 21 effective single sequences, as shown in Table 6. The single sequences generated in the first discussion mainly include identification and approval point → analysis and explanation point (A4→A5), and there are four sequences in total.

**Table 6:** Behavioral adjustment residual values of critical thinking process in the high engagement group.

**Given:**	**R1**	**R2**	**U1**	**U2**	**U3**	**U4**	**U5**	**U6**	**A1**	**A2**	**A3**	**A4**	**A5**	**A6**	**E1**	**E2**	**E3**	**E4**	**E5**	**E6**	**C1**	**C2**	**C3**	**N1**	**N2**	**Totals**
R1	0	0	0	0	0	0	1	0	0	0	1	1	0	0	0	0	0	0	0	0	0	0	0	0	0	3
R2	1	0	0	0	0	1	0	0	0	0	0	4	0	0	0	0	0	0	1	0	0	0	0	0	0	7
U1	0	0	0	0	0	0	0	0	0	0	0	0	0	0	0	0	0	0	0	0	0	0	0	0	0	0
U2	0	0	0	0	0	0	0	0	0	0	0	0	0	0	0	1	0	0	0	0	0	0	0	0	0	1
U3	0	0	0	0	0	0	0	0	0	1	0	0	0	0	0	0	0	0	0	0	0	0	0	0	0	1
U4	0	0	0	0	0	0	0	0	0	0	0	0	0	0	0	0	0	0	0	0	0	0	0	0	0	0
U5	1	1	0	0	0	0	0	0	0	1	0	1	0	0	0	0	0	0	0	0	0	0	0	0	0	4
U6	0	0	0	0	0	0	0	0	0	0	0	0	0	0	0	0	0	0	0	0	0	0	0	0	0	0
A1	0	0	0	0	0	0	0	0	0	1	0	1	0	0	0	0	0	0	0	0	0	0	0	0	0	2
A2	0	0	0	0	0	0	0	0	0	0	0	1	0	0	0	0	0	0	0	0	0	0	0	0	0	1
A3	0	0	0	0	0	0	0	0	0	0	0	0	0	0	0	0	0	0	0	0	0	0	0	0	0	0
A4	0	0	0	0	0	0	0	0	0	0	0	0	1	0	0	0	0	0	0	0	0	0	0	0	0	1
A5	0	0	0	0	0	0	0	0	0	0	0	0	0	0	0	0	0	0	0	0	0	0	0	0	0	0
A6	0	0	0	0	0	0	0	0	0	0	0	0	0	0	0	0	0	0	0	0	0	0	0	0	0	0
E1	0	0	0	0	0	0	0	0	0	0	0	0	0	0	0	0	0	0	0	0	0	0	0	0	0	0
E2	0	1	0	0	0	0	0	0	0	0	0	0	0	0	0	0	0	0	0	0	0	0	0	0	0	1
E3	0	0	0	0	0	0	0	0	0	0	0	0	0	0	0	0	0	0	0	0	0	0	0	0	0	0
E4	0	0	0	0	0	0	0	0	0	0	0	0	0	0	0	0	0	0	0	0	0	0	0	0	0	0
E5	0	0	0	0	0	0	0	0	0	0	0	0	0	0	0	0	0	0	0	0	0	0	0	0	0	0
E6	0	0	0	0	0	0	0	0	0	0	0	0	0	0	0	0	0	0	0	0	0	0	0	0	0	0
C1	0	0	0	0	0	0	0	0	0	0	0	0	0	0	0	0	0	0	0	0	0	0	0	0	0	0
C2	0	0	0	0	0	0	0	0	0	0	0	0	0	0	0	0	0	0	0	0	0	0	0	0	0	0
C3	0	0	0	0	0	0	0	0	0	0	0	0	0	0	0	0	0	0	0	0	0	0	0	0	0	0
N1	0	0	0	0	0	0	0	0	0	0	0	0	0	0	0	0	0	0	0	0	0	0	0	0	0	0
N2	0	0	0	0	0	0	0	0	0	0	0	0	0	0	0	0	0	0	0	0	0	0	0	0	0	0
Totals	2	2	0	0	0	1	1	0	0	3	1	8	1	0	0	1	0	0	1	0	0	0	0	0	0	21

**Figure 3:**
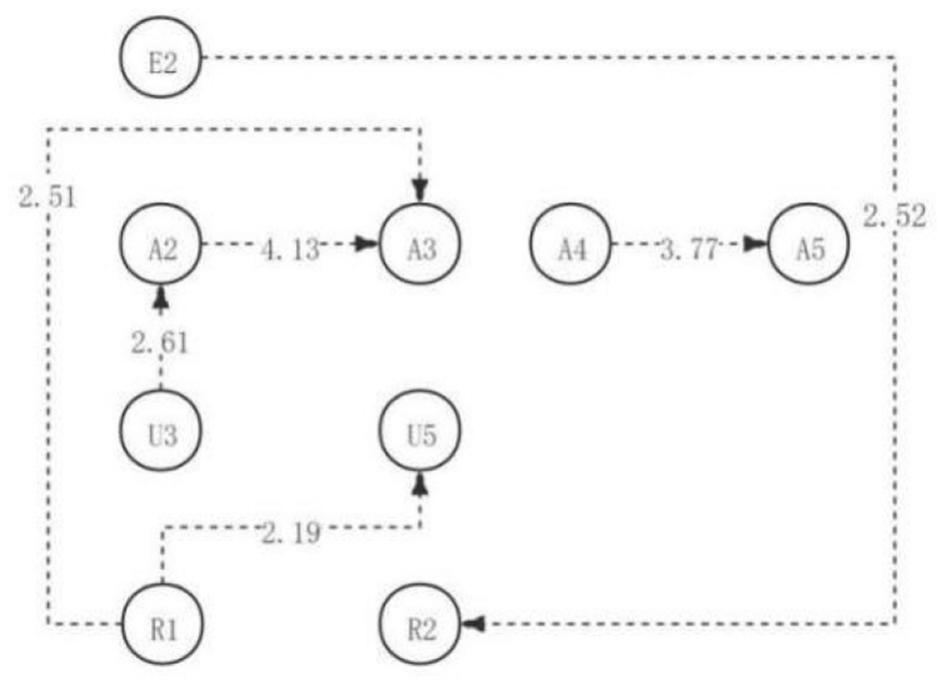
The critical thinking discussion activity path diagram of the high engagement group.

Among them, there are five significant sequences with a residual value >1.96 (as shown in Table 7). Identifying and reiterating questions → understanding and answering questions (R1→U5), identifying and reiterating questions → analyzing similarities and differences of viewpoints (R1→A3), searching for different evidence → classification and classification of evidence (U3→A2), analyzing and explaining viewpoints → decomposition of viewpoints (A4→A5), and criticizing assumptions → identifying and approving questions (E2→R2). In addition, according to the adjusted sequence of significant behavioral residuals, a complete directed path diagram of learning activities is generated, as shown in Figure 3. As can be seen from the visual path diagram of the first discussion, after the initial identification of the problem, some students in the high engagement group proceeded to evaluate and clarify the problem to explore its essence (R1→U5), while some students proceeded to analyze the similarities and differences of various viewpoints (R1→A3). After looking for different pieces of evidence, students will further classify the collected evidence (U3→A2). After analyzing and explaining ideas, students will further decompose their ideas (A4→A5). Students criticize the opinion, but at the same time, they put forward the part that they agree with (E2→R2).

**Table 7:** Behavioral adjustment residual values of critical thinking process in the high engagement group.

**Given:**	**R1**	**R2**	**U1**	**U2**	**U3**	**U4**	**U5**	**U6**	**A1**	**A2**	**A3**	**A4**	**A5**	**A6**	**E1**	**E2**	**E3**	**E4**	**E5**	**E6**	**C1**	**C2**	**C3**	**N1**	**N2**
R1	0	−0.75	0	0	0	−0.42	2.19^*^	0	0	−0.78	2.51^*^	−0.28	−0.42	0	0	−0.43	0	0	−0.42	0	0	0	0	0	0
R2	0.28	0	0	0	0	1.4	−0.81	0	0	−1.38	−0.74	0.95	−0.74	0	0	−0.75	0	0	1.4	0	0	0	0	0	0
U1	0	0	0	0	0	0	0	0	0	0	0	0	0	0	0	0	0	0	0	0	0	0	0	0	0
U2	−0.34	−0.39	0	0	0	−0.22	−0.24	0	0	−0.41	−0.22	−0.79	−0.22	0	0	4.74^*^	0	0	−0.22	0	0	0	0	0	0
U3	−0.34	−0.39	0	0	0	−0.22	−0.24	0	0	2.61^*^	−0.22	−0.79	−0.22	0	0	−0.22	0	0	−0.22	0	0	0	0	0	0
U4	0	0	0	0	0	0	0	0	0	0	0	0	0	0	0	0	0	0	0	0	0	0	0	0	0
U5	1.04	0.71	0	0	0	−0.48	0	0	0	0.68	−0.48	−0.62	−0.48	0	0	−0.5	0	0	−0.48	0	0	0	0	0	0
U6	0	0	0	0	0	0	0	0	0	0	0	0	0	0	0	0	0	0	0	0	0	0	0	0	0
A1	−0.49	−0.56	0	0	0	−0.32	−0.35	0	0	1.6	−0.32	0.42	−0.32	0	0	−0.32	0	0	−0.32	0	0	0	0	0	0
A2	−0.37	−0.42	0	0	0	−0.23	−0.26	0	0	0	−0.23	1.15	−0.23	0	0	−0.24	0	0	−0.23	0	0	0	0	0	0
A3	0	0	0	0	0	0	0	0	0	0	0	0	0	0	0	0	0	0	0	0	0	0	0	0	0
A4	−0.43	−0.49	0	0	0	−0.27	−0.3	0	0	−0.51	−0.27	0	3.77^*^	0	0	−0.28	0	0	−0.27	0	0	0	0	0	0
A5	0	0	0	0	0	0	0	0	0	0	0	0	0	0	0	0	0	0	0	0	0	0	0	0	0
A6	0	0	0	0	0	0	0	0	0	0	0	0	0	0	0	0	0	0	0	0	0	0	0	0	0
E1	0	0	0	0	0	0	0	0	0	0	0	0	0	0	0	0	0	0	0	0	0	0	0	0	0
E2	−0.35	2.52^*^	0	0	0	−0.22	−0.25	0	0	−0.42	−0.22	−0.81	−0.22	0	0	0	0	0	−0.22	0	0	0	0	0	0
E3	0	0	0	0	0	0	0	0	0	0	0	0	0	0	0	0	0	0	0	0	0	0	0	0	0
E4	0	0	0	0	0	0	0	0	0	0	0	0	0	0	0	0	0	0	0	0	0	0	0	0	0
E5	0	0	0	0	0	0	0	0	0	0	0	0	0	0	0	0	0	0	0	0	0	0	0	0	0
E6	0	0	0	0	0	0	0	0	0	0	0	0	0	0	0	0	0	0	0	0	0	0	0	0	0
C1	0	0	0	0	0	0	0	0	0	0	0	0	0	0	0	0	0	0	0	0	0	0	0	0	0
C2	0	0	0	0	0	0	0	0	0	0	0	0	0	0	0	0	0	0	0	0	0	0	0	0	0
C3	0	0	0	0	0	0	0	0	0	0	0	0	0	0	0	0	0	0	0	0	0	0	0	0	0
N1	0	0	0	0	0	0	0	0	0	0	0	0	0	0	0	0	0	0	0	0	0	0	0	0	0
N2	0	0	0	0	0	0	0	0	0	0	0	0	0	0	0	0	0	0	0	0	0	0	0	0	0

* *Indicates that the Z-score value is significant*.

**Figure 4:**
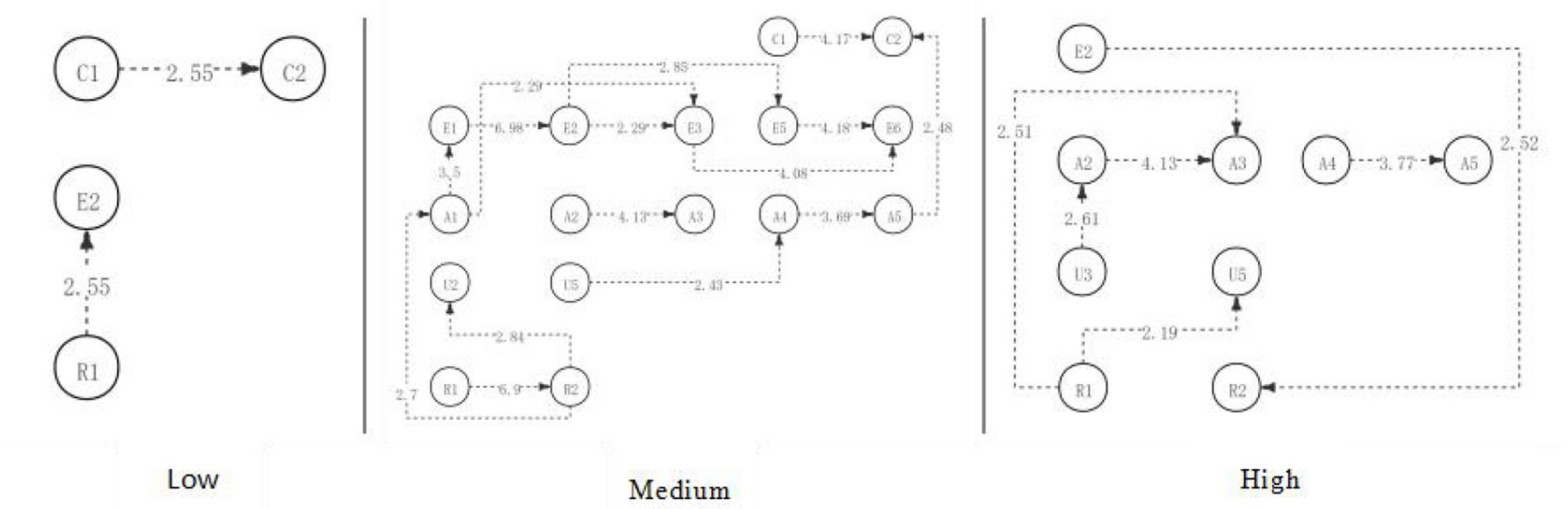
The critical thinking discussion activity path diagram of the different engagement group.

The results above indicated that, to sum up, in the first discussion, students with high, medium, and low academic engagement showed significant differences in the visualized critical thinking activity path diagrams in the discussion activities (as shown in Figure 4). However, contrary to the expectations, students in both the low and medium engagement groups reached the highest level of critical thinking—creativity; students in the high engagement group only reached the evaluation level. In contrast to the previous hypothesis, there were more significant behavioral sequences in the medium engagement group than in the high engagement group. The significant behavior sequence of critical thinking activities of students in the high and medium engagement groups was more than that in the low engagement group, which was consistent with the hypothesis of the study.

### Critical Thinking Pathways of Participants With Different Levels of Academic Engagement After the Second STEAM Course

According to the coding results, the characteristics of critical thinking pathways of students in low, medium, and high academic engagement groups were analyzed. The specific results are analyzed as follows.

For students with low academic engagement, only one effective single sequence U5→A4 is generated at this stage, as shown in Table 8. However, there was no significant behavioral residual (as shown in Table 9).

**Table 8:** Behavioral adjustment residual values of critical thinking process in the low engagement group.

**Given:**	**R1**	**R2**	**U1**	**U2**	**U3**	**U4**	**U5**	**U6**	**A1**	**A2**	**A3**	**A4**	**A5**	**A6**	**E1**	**E2**	**E3**	**E4**	**E5**	**E6**	**C1**	**C2**	**C3**	**N1**	**N2**	**Totals**
R1	0	0	0	0	0	0	0	0	0	0	0	0	0	0	0	0	0	0	0	0	0	0	0	0	0	0
R2	0	0	0	0	0	0	0	0	0	0	0	0	0	0	0	0	0	0	0	0	0	0	0	0	0	0
U1	0	0	0	0	0	0	0	0	0	0	0	0	0	0	0	0	0	0	0	0	0	0	0	0	0	0
U2	0	0	0	0	0	0	0	0	0	0	0	0	0	0	0	0	0	0	0	0	0	0	0	0	0	0
U3	0	0	0	0	0	0	0	0	0	0	0	0	0	0	0	0	0	0	0	0	0	0	0	0	0	0
U4	0	0	0	0	0	0	0	0	0	0	0	0	0	0	0	0	0	0	0	0	0	0	0	0	0	0
U5	0	0	0	0	0	0	0	0	0	0	0	1	0	0	0	0	0	0	0	0	0	0	0	0	0	1
U6	0	0	0	0	0	0	0	0	0	0	0	0	0	0	0	0	0	0	0	0	0	0	0	0	0	0
A1	0	0	0	0	0	0	0	0	0	0	0	0	0	0	0	0	0	0	0	0	0	0	0	0	0	0
A2	0	0	0	0	0	0	0	0	0	0	0	0	0	0	0	0	0	0	0	0	0	0	0	0	0	0
A3	0	0	0	0	0	0	0	0	0	0	0	0	0	0	0	0	0	0	0	0	0	0	0	0	0	0
A4	0	0	0	0	0	0	0	0	0	0	0	0	0	0	0	0	0	0	0	0	0	0	0	0	0	0
A5	0	0	0	0	0	0	0	0	0	0	0	0	0	0	0	0	0	0	0	0	0	0	0	0	0	0
A6	0	0	0	0	0	0	0	0	0	0	0	0	0	0	0	0	0	0	0	0	0	0	0	0	0	0
E1	0	0	0	0	0	0	0	0	0	0	0	0	0	0	0	0	0	0	0	0	0	0	0	0	0	0
E2	0	0	0	0	0	0	0	0	0	0	0	0	0	0	0	0	0	0	0	0	0	0	0	0	0	0
E3	0	0	0	0	0	0	0	0	0	0	0	0	0	0	0	0	0	0	0	0	0	0	0	0	0	0
E4	0	0	0	0	0	0	0	0	0	0	0	0	0	0	0	0	0	0	0	0	0	0	0	0	0	0
E5	0	0	0	0	0	0	0	0	0	0	0	0	0	0	0	0	0	0	0	0	0	0	0	0	0	0
E6	0	0	0	0	0	0	0	0	0	0	0	0	0	0	0	0	0	0	0	0	0	0	0	0	0	0
C1	0	0	0	0	0	0	0	0	0	0	0	0	0	0	0	0	0	0	0	0	0	0	0	0	0	0
C2	0	0	0	0	0	0	0	0	0	0	0	0	0	0	0	0	0	0	0	0	0	0	0	0	0	0
C3	0	0	0	0	0	0	0	0	0	0	0	0	0	0	0	0	0	0	0	0	0	0	0	0	0	0
N1	0	0	0	0	0	0	0	0	0	0	0	0	0	0	0	0	0	0	0	0	0	0	0	0	0	0
N2	0	0	0	0	0	0	0	0	0	0	0	0	0	0	0	0	0	0	0	0	0	0	0	0	0	0
Totals	0	0	0	0	0	0	0	0	0	0	0	1	0	0	0	0	0	0	0	0	0	0	0	0	0	1

**Table 9:** Behavioral adjustment residual values of critical thinking process in the low engagement group.

**Given:**	**R1**	**R2**	**U1**	**U2**	**U3**	**U4**	**U5**	**U6**	**A1**	**A2**	**A3**	**A4**	**A5**	**A6**	**E1**	**E2**	**E3**	**E4**	**E5**	**E6**	**C1**	**C2**	**C3**	**N1**	**N2**
R1	0	0	0	0	0	0	0	0	0	0	0	0	0	0	0	0	0	0	0	0	0	0	0	0	0
R2	0	0	0	0	0	0	0	0	0	0	0	0	0	0	0	0	0	0	0	0	0	0	0	0	0
U1	0	0	0	0	0	0	0	0	0	0	0	0	0	0	0	0	0	0	0	0	0	0	0	0	0
U2	0	0	0	0	0	0	0	0	0	0	0	0	0	0	0	0	0	0	0	0	0	0	0	0	0
U3	0	0	0	0	0	0	0	0	0	0	0	0	0	0	0	0	0	0	0	0	0	0	0	0	0
U4	0	0	0	0	0	0	0	0	0	0	0	0	0	0	0	0	0	0	0	0	0	0	0	0	0
U5	0	0	0	0	0	0	0	0	0	0	0	0	0	0	0	0	0	0	0	0	0	0	0	0	0
U6	0	0	0	0	0	0	0	0	0	0	0	0	0	0	0	0	0	0	0	0	0	0	0	0	0
A1	0	0	0	0	0	0	0	0	0	0	0	0	0	0	0	0	0	0	0	0	0	0	0	0	0
A2	0	0	0	0	0	0	0	0	0	0	0	0	0	0	0	0	0	0	0	0	0	0	0	0	0
A3	0	0	0	0	0	0	0	0	0	0	0	0	0	0	0	0	0	0	0	0	0	0	0	0	0
A4	0	0	0	0	0	0	0	0	0	0	0	0	0	0	0	0	0	0	0	0	0	0	0	0	0
A5	0	0	0	0	0	0	0	0	0	0	0	0	0	0	0	0	0	0	0	0	0	0	0	0	0
A6	0	0	0	0	0	0	0	0	0	0	0	0	0	0	0	0	0	0	0	0	0	0	0	0	0
E1	0	0	0	0	0	0	0	0	0	0	0	0	0	0	0	0	0	0	0	0	0	0	0	0	0
E2	0	0	0	0	0	0	0	0	0	0	0	0	0	0	0	0	0	0	0	0	0	0	0	0	0
E3	0	0	0	0	0	0	0	0	0	0	0	0	0	0	0	0	0	0	0	0	0	0	0	0	0
E4	0	0	0	0	0	0	0	0	0	0	0	0	0	0	0	0	0	0	0	0	0	0	0	0	0
E5	0	0	0	0	0	0	0	0	0	0	0	0	0	0	0	0	0	0	0	0	0	0	0	0	0
E6	0	0	0	0	0	0	0	0	0	0	0	0	0	0	0	0	0	0	0	0	0	0	0	0	0
C1	0	0	0	0	0	0	0	0	0	0	0	0	0	0	0	0	0	0	0	0	0	0	0	0	0
C2	0	0	0	0	0	0	0	0	0	0	0	0	0	0	0	0	0	0	0	0	0	0	0	0	0
C3	0	0	0	0	0	0	0	0	0	0	0	0	0	0	0	0	0	0	0	0	0	0	0	0	0
N1	0	0	0	0	0	0	0	0	0	0	0	0	0	0	0	0	0	0	0	0	0	0	0	0	0
N2	0	0	0	0	0	0	0	0	0	0	0	0	0	0	0	0	0	0	0	0	0	0	0	0	0

For students with medium academic engagement, 12 kinds and 18 effective single sequences are generated in this stage, such as U2→A2, R1→U2, R2→R1, A2→C1, as shown in Table 10. The second discussion produced more single sequences mainly for understanding and seeking relevant content → evidence classification and classification (U2→A2). There were four sequences in total and relatively few sequences from the process of creating (C).

**Table 10:** Behavioral adjustment residual values of critical thinking process in the medium engagement group.

**Given:**	**R1**	**R2**	**U1**	**U2**	**U3**	**U4**	**U5**	**U6**	**A1**	**A2**	**A3**	**A4**	**A5**	**A6**	**E1**	**E2**	**E3**	**E4**	**E5**	**E6**	**C1**	**C2**	**C3**	**N1**	**N2**	**Totals**
R1	0	0	0	2	0	0	0	0	0	0	0	0	0	0	0	0	0	0	0	0	0	0	0	0	0	2
R2	2	0	0	0	0	0	1	0	1	1	0	1	0	0	0	0	0	0	1	0	0	0	0	0	0	7
U1	0	0	0	0	0	0	0	0	0	0	0	0	0	0	0	0	0	0	0	0	0	0	0	0	0	0
U2	0	0	0	0	0	0	0	0	0	4	1	0	0	0	0	0	0	0	0	0	0	0	0	0	0	5
U3	0	0	0	0	0	0	0	0	0	0	0	0	0	0	0	0	0	0	0	0	0	0	0	0	0	0
U4	0	0	0	0	0	0	0	0	0	0	0	0	0	0	0	0	0	0	0	0	0	0	0	0	0	0
U5	0	0	0	0	0	0	0	0	0	0	0	0	0	0	0	0	0	0	0	0	0	0	0	0	0	0
U6	0	0	0	0	0	0	0	0	0	0	0	0	0	0	0	0	0	0	0	0	0	0	0	0	0	0
A1	0	0	0	0	0	0	0	0	0	0	0	0	0	0	0	0	0	0	0	0	0	0	0	0	0	0
A2	0	0	0	0	0	0	0	0	0	0	0	0	0	0	0	0	0	0	0	0	2	0	0	0	0	2
A3	0	0	0	0	0	0	0	0	0	0	0	0	0	0	0	0	0	0	0	0	0	0	0	0	0	0
A4	0	0	0	0	0	0	0	0	0	0	0	0	1	0	0	0	0	0	0	0	0	0	0	0	0	1
A5	0	0	0	0	0	0	0	0	0	0	0	0	0	0	0	0	0	0	0	0	0	0	0	0	0	0
A6	0	0	0	0	0	0	0	0	0	0	0	0	0	0	0	0	0	0	0	0	0	0	0	0	0	0
E1	0	0	0	0	0	0	0	0	0	0	0	0	0	0	0	0	0	0	0	0	0	0	0	0	0	0
E2	0	0	0	0	0	0	0	0	0	0	0	0	0	0	0	0	0	0	0	0	0	0	0	0	0	0
E3	0	0	0	0	0	0	0	0	0	0	0	0	0	0	0	0	0	0	0	0	0	0	0	0	0	0
E4	0	0	0	0	0	0	0	0	0	0	0	0	0	0	0	0	0	0	0	0	0	0	0	0	0	0
E5	1	0	0	0	0	0	0	0	0	0	0	0	0	0	0	0	0	0	0	0	0	0	0	0	0	1
E6	0	0	0	0	0	0	0	0	0	0	0	0	0	0	0	0	0	0	0	0	0	0	0	0	0	0
C1	0	0	0	0	0	0	0	0	0	0	0	0	0	0	0	0	0	0	0	0	0	0	0	0	0	0
C2	0	0	0	0	0	0	0	0	0	0	0	0	0	0	0	0	0	0	0	0	0	0	0	0	0	0
C3	0	0	0	0	0	0	0	0	0	0	0	0	0	0	0	0	0	0	0	0	0	0	0	0	0	0
N1	0	0	0	0	0	0	0	0	0	0	0	0	0	0	0	0	0	0	0	0	0	0	0	0	0	0
N2	0	0	0	0	0	0	0	0	0	0	0	0	0	0	0	0	0	0	0	0	0	0	0	0	0	0
Totals	3	0	0	2	0	0	1	0	1	5	1	1	1	0	0	0	0	0	1	0	2	0	0	0	0	18

**Figure 5:**
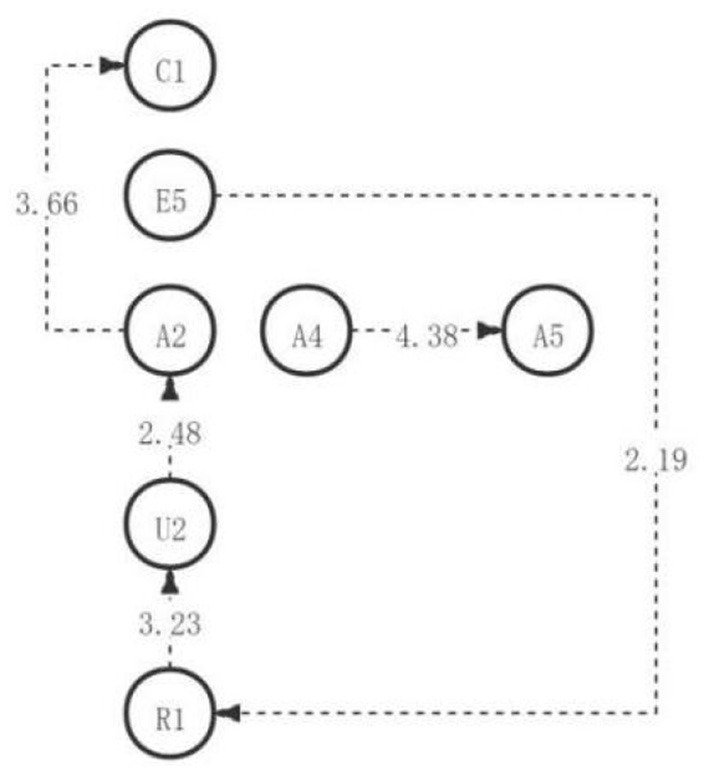
The critical thinking discussion activity path diagram of the medium engagement group.

Among them, there are five significant sequences with a residual value >1.96 (as shown in Table 11). That is, to identify and reiterate the problem → understand and search for relevant content (R1→U2), understand and search for relevant content → classification and classification of evidence (U2→A2), classification and classification of evidence → application and implementation plan (A2→C1), analysis and explanation of viewpoints → decomposition of problem viewpoints (A4→A5), and supporting arguments → identification and reiterate the problem (E5→R1). According to the adjusted sequence of significant behavioral residuals, a complete directed path diagram of learning activities is generated, as shown in Figure 5. As can be seen from the visual path diagram of the second discussion, students in the input group initially identified the problem, then searched for some contents related to the problem, and then classified these contents. Finally, based on this, they implemented the new plans and ideas according to the requirements of the problem (R1→U2→A2→C1). After analyzing the current opinion, students will usually further break down the current opinion or the problem (A4→A5). After using evidence to support the argument, students will further reiterate the problem to be solved (E5→R1).

**Table 11:** Behavioral adjustment residual values of critical thinking process in the medium engagement group.

**Given:**	**R1**	**R2**	**U1**	**U2**	**U3**	**U4**	**U5**	**U6**	**A1**	**A2**	**A3**	**A4**	**A5**	**A6**	**E1**	**E2**	**E3**	**E4**	**E5**	**E6**	**C1**	**C2**	**C3**	**N1**	**N2**
R1	0	0	0	3.23^*^	0	0	−0.38	0	−0.38	−1.04	−0.38	−0.39	−0.38	0	0	0	0	0	−0.39	0	−0.55	0	0	0	0
R2	1.05	0	0	−1.34	0	0	1.46	0	1.46	−1.08	−0.78	1.38	−0.78	0	0	0	0	0	1.38	0	−1.13	0	0	0	0
U1	0	0	0	0	0	0	0	0	0	0	0	0	0	0	0	0	0	0	0	0	0	0	0	0	0
U2	−1.28	0	0	0	0	0	−0.65	0	−0.65	2.48^*^	1.6	−0.67	−0.65	0	0	0	0	0	−0.67	0	−0.95	0	0	0	0
U3	0	0	0	0	0	0	0	0	0	0	0	0	0	0	0	0	0	0	0	0	0	0	0	0	0
U4	0	0	0	0	0	0	0	0	0	0	0	0	0	0	0	0	0	0	0	0	0	0	0	0	0
U5	0	0	0	0	0	0	0	0	0	0	0	0	0	0	0	0	0	0	0	0	0	0	0	0	0
U6	0	0	0	0	0	0	0	0	0	0	0	0	0	0	0	0	0	0	0	0	0	0	0	0	0
A1	0	0	0	0	0	0	0	0	0	0	0	0	0	0	0	0	0	0	0	0	0	0	0	0	0
A2	−0.8	0	0	−0.7	0	0	−0.41	0	−0.41	0	−0.41	−0.42	−0.41	0	0	0	0	0	−0.42	0	3.66^*^	0	0	0	0
A3	0	0	0	0	0	0	0	0	0	0	0	0	0	0	0	0	0	0	0	0	0	0	0	0	0
A4	−0.48	0	0	−0.42	0	0	−0.24	0	−0.24	−0.67	−0.24	0	4.38^*^	0	0	0	0	0	−0.25	0	−0.35	0	0	0	0
A5	0	0	0	0	0	0	0	0	0	0	0	0	0	0	0	0	0	0	0	0	0	0	0	0	0
A6	0	0	0	0	0	0	0	0	0	0	0	0	0	0	0	0	0	0	0	0	0	0	0	0	0
E1	0	0	0	0	0	0	0	0	0	0	0	0	0	0	0	0	0	0	0	0	0	0	0	0	0
E2	0	0	0	0	0	0	0	0	0	0	0	0	0	0	0	0	0	0	0	0	0	0	0	0	0
E3	0	0	0	0	0	0	0	0	0	0	0	0	0	0	0	0	0	0	0	0	0	0	0	0	0
E4	0	0	0	0	0	0	0	0	0	0	0	0	0	0	0	0	0	0	0	0	0	0	0	0	0
E5	2.19^*^	0	0	−0.42	0	0	−0.24	0	−0.24	−0.67	−0.24	−0.25	−0.24	0	0	0	0	0	0	0	−0.35	0	0	0	0
E6	0	0	0	0	0	0	0	0	0	0	0	0	0	0	0	0	0	0	0	0	0	0	0	0	0
C1	0	0	0	0	0	0	0	0	0	0	0	0	0	0	0	0	0	0	0	0	0	0	0	0	0
C2	0	0	0	0	0	0	0	0	0	0	0	0	0	0	0	0	0	0	0	0	0	0	0	0	0
C3	0	0	0	0	0	0	0	0	0	0	0	0	0	0	0	0	0	0	0	0	0	0	0	0	0
N1	0	0	0	0	0	0	0	0	0	0	0	0	0	0	0	0	0	0	0	0	0	0	0	0	0
N2	0	0	0	0	0	0	0	0	0	0	0	0	0	0	0	0	0	0	0	0	0	0	0	0	0

* *Indicates that the Z–score value is significant*.

For students with high academic engagement, there are altogether eight kinds and 12 effective single sequences, such as R2→A1, A2→C1, in this stage, as shown in Table 12. In the second discussion, more single sequences were generated, mainly from the evidence classification → application implementation plan (A2→C1), with a total of four sequences, and relatively fewer from process sequences to creation (C).

**Table 12:** Behavioral adjustment residual values of critical thinking process in the high engagement group.

**Given:**	**R1**	**R2**	**U1**	**U2**	**U3**	**U4**	**U5**	**U6**	**A1**	**A2**	**A3**	**A4**	**A5**	**A6**	**E1**	**E2**	**E3**	**E4**	**E5**	**E6**	**C1**	**C2**	**C3**	**N1**	**N2**	**Totals**
R1	0	0	0	0	0	0	0	0	0	1	0	0	0	0	0	0	0	0	0	0	0	0	0	0	0	1
R2	0	0	0	1	0	0	1	0	2	0	0	0	0	0	0	0	0	0	0	0	0	0	0	0	0	4
U1	0	0	0	0	0	0	0	0	0	0	0	0	0	0	0	0	0	0	0	0	0	0	0	0	0	0
U2	1	0	0	0	0	0	0	0	0	0	0	0	0	0	0	0	0	0	0	0	0	0	0	0	0	1
U3	0	0	0	0	0	0	0	0	0	0	0	0	0	0	0	0	0	0	0	0	0	0	0	0	0	0
U4	0	0	0	0	0	0	0	0	0	0	0	0	0	0	0	0	0	0	0	0	0	0	0	0	0	0
U5	0	0	0	0	0	0	0	0	0	0	0	0	0	0	0	1	0	0	0	0	0	0	0	0	0	1
U6	0	0	0	0	0	0	0	0	0	0	0	0	0	0	0	0	0	0	0	0	0	0	0	0	0	0
A1	0	0	0	0	0	0	0	0	0	0	0	0	0	0	0	0	0	0	0	0	0	0	0	0	0	0
A2	0	0	0	0	0	0	0	0	0	0	0	0	0	0	0	0	0	0	0	0	4	0	0	0	0	4
A3	0	0	0	0	0	0	0	0	0	0	0	0	0	0	0	0	0	0	0	0	0	0	0	0	0	0
A4	0	0	0	0	0	0	0	0	0	0	0	0	1	0	0	0	0	0	0	0	0	0	0	0	0	1
A5	0	0	0	0	0	0	0	0	0	0	0	0	0	0	0	0	0	0	0	0	0	0	0	0	0	0
A6	0	0	0	0	0	0	0	0	0	0	0	0	0	0	0	0	0	0	0	0	0	0	0	0	0	0
E1	0	0	0	0	0	0	0	0	0	0	0	0	0	0	0	0	0	0	0	0	0	0	0	0	0	0
E2	0	0	0	0	0	0	0	0	0	0	0	0	0	0	0	0	0	0	0	0	0	0	0	0	0	0
E3	0	0	0	0	0	0	0	0	0	0	0	0	0	0	0	0	0	0	0	0	0	0	0	0	0	0
E4	0	0	0	0	0	0	0	0	0	0	0	0	0	0	0	0	0	0	0	0	0	0	0	0	0	0
E5	0	0	0	0	0	0	0	0	0	0	0	0	0	0	0	0	0	0	0	0	0	0	0	0	0	0
E6	0	0	0	0	0	0	0	0	0	0	0	0	0	0	0	0	0	0	0	0	0	0	0	0	0	0
C1	0	0	0	0	0	0	0	0	0	0	0	0	0	0	0	0	0	0	0	0	0	0	0	0	0	0
C2	0	0	0	0	0	0	0	0	0	0	0	0	0	0	0	0	0	0	0	0	0	0	0	0	0	0
C3	0	0	0	0	0	0	0	0	0	0	0	0	0	0	0	0	0	0	0	0	0	0	0	0	0	0
N1	0	0	0	0	0	0	0	0	0	0	0	0	0	0	0	0	0	0	0	0	0	0	0	0	0	0
N2	0	0	0	0	0	0	0	0	0	0	0	0	0	0	0	0	0	0	0	0	0	0	0	0	0	0
Totals	1	0	0	1	0	0	1	0	2	1	0	0	1	0	0	1	0	0	0	0	4	0	0	0	0	12

**Figure 6:**
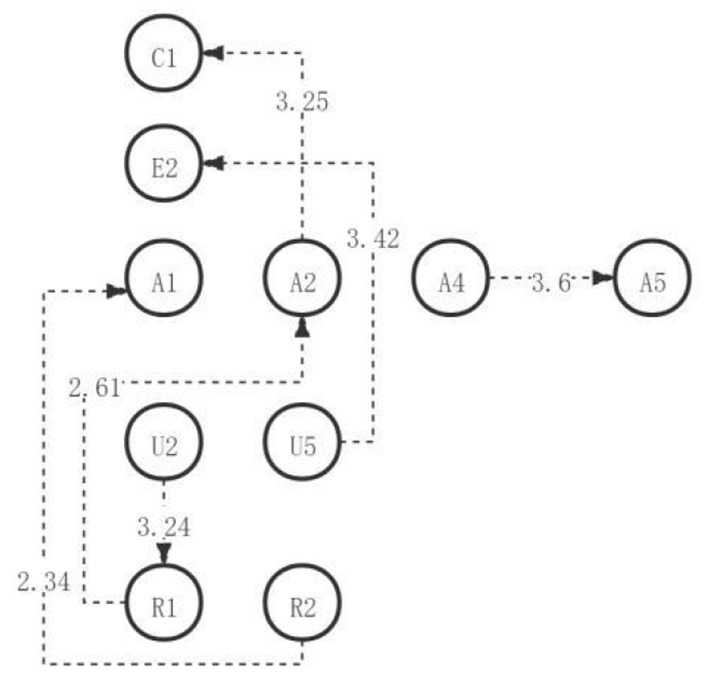
The critical thinking discussion activity path diagram of the high engagement group.

Among them, there are six significant sequences with a residual value >1.96 (as shown in Table 13). That is, to identify and restate the problem → evidence classification and classification (R1→A2), identify and approve the problem → add new thinking behavior (R2→A1), understand and find relevant content → identify and restate the problem (U2→R1), understand and answer the problem → critical hypothesis (U5→E2), evidence classification and classification → application implementation plan (A2→C1), analyze and explain the point of view → problem view decompose (A4→A5). According to the adjusted sequence of significant behavioral residuals, a complete directed path diagram of learning activities is generated, as shown in Figure 6. As can be seen from the visual path diagram of the second discussion, students in the high engagement group initially identified the problem, then classified the evidence related to the problem, and directly put the new plan and new idea into practice on this basis (R1→A2→C1). Based on identifying the approval problem, students add new thinking or new behavior mode (R2→A1). After students understand the problem and look for relevant content, they further identify and reiterate the problem (U2→R1). After the students understand and answer the questions, they further criticize the current opinions (U5→E2). During the discussion, students usually breakdown the problem further (A4→A5) after explaining the current problem.

**Table 13:** Behavioral adjustment residual values of critical thinking process in the high engagement group.

**Given:**	**R1**	**R2**	**U1**	**U2**	**U3**	**U4**	**U5**	**U6**	**A1**	**A2**	**A3**	**A4**	**A5**	**A6**	**E1**	**E2**	**E3**	**E4**	**E5**	**E6**	**C1**	**C2**	**C3**	**N1**	**N2**
R1	0	0	0	−0.33	0	0	−0.33	0	−0.47	2.61	0	0	−0.32	0	0	−0.32	0	0	0	0	−0.75	0	0	0	0
R2	−0.75	0	0	1.44	0	0	1.44	0	2.34	−0.89	0	0	−0.71	0	0	−0.71	0	0	0	0	−1.68	0	0	0	0
U1	0	0	0	0	0	0	0	0	0	0	0	0	0	0	0	0	0	0	0	0	0	0	0	0	0
U2	3.24^*^	0	0	0	0	0	−0.33	0	−0.47	−0.4	0	0	−0.32	0	0	−0.32	0	0	0	0	−0.75	0	0	0	0
U3	0	0	0	0	0	0	0	0	0	0	0	0	0	0	0	0	0	0	0	0	0	0	0	0	0
U4	0	0	0	0	0	0	0	0	0	0	0	0	0	0	0	0	0	0	0	0	0	0	0	0	0
U5	−0.33	0	0	−0.33	0	0	0	0	−0.47	−0.4	0	0	−0.32	0	0	3.42^*^	0	0	0	0	−0.75	0	0	0	0
U6	0	0	0	0	0	0	0	0	0	0	0	0	0	0	0	0	0	0	0	0	0	0	0	0	0
A1	0	0	0	0	0	0	0	0	0	0	0	0	0	0	0	0	0	0	0	0	0	0	0	0	0
A2	−0.8	0	0	−0.8	0	0	−0.8	0	−1.13	0	0	0	−0.76	0	0	−0.76	0	0	0	0	3.25^*^	0	0	0	0
A3	0	0	0	0	0	0	0	0	0	0	0	0	0	0	0	0	0	0	0	0	0	0	0	0	0
A4	−0.32	0	0	−0.32	0	0	−0.32	0	−0.45	−0.38	0	0	3.6^*^	0	0	−0.3	0	0	0	0	−0.71	0	0	0	0
A5	0	0	0	0	0	0	0	0	0	0	0	0	0	0	0	0	0	0	0	0	0	0	0	0	0
A6	0	0	0	0	0	0	0	0	0	0	0	0	0	0	0	0	0	0	0	0	0	0	0	0	0
E1	0	0	0	0	0	0	0	0	0	0	0	0	0	0	0	0	0	0	0	0	0	0	0	0	0
E2	0	0	0	0	0	0	0	0	0	0	0	0	0	0	0	0	0	0	0	0	0	0	0	0	0
E3	0	0	0	0	0	0	0	0	0	0	0	0	0	0	0	0	0	0	0	0	0	0	0	0	0
E4	0	0	0	0	0	0	0	0	0	0	0	0	0	0	0	0	0	0	0	0	0	0	0	0	0
E5	0	0	0	0	0	0	0	0	0	0	0	0	0	0	0	0	0	0	0	0	0	0	0	0	0
E6	0	0	0	0	0	0	0	0	0	0	0	0	0	0	0	0	0	0	0	0	0	0	0	0	0
C1	0	0	0	0	0	0	0	0	0	0	0	0	0	0	0	0	0	0	0	0	0	0	0	0	0
C2	0	0	0	0	0	0	0	0	0	0	0	0	0	0	0	0	0	0	0	0	0	0	0	0	0
C3	0	0	0	0	0	0	0	0	0	0	0	0	0	0	0	0	0	0	0	0	0	0	0	0	0
N1	0	0	0	0	0	0	0	0	0	0	0	0	0	0	0	0	0	0	0	0	0	0	0	0	0
N2	0	0	0	0	0	0	0	0	0	0	0	0	0	0	0	0	0	0	0	0	0	0	0	0	0

* *Indicates that the Z-score value is significant*.

**Figure 7:**
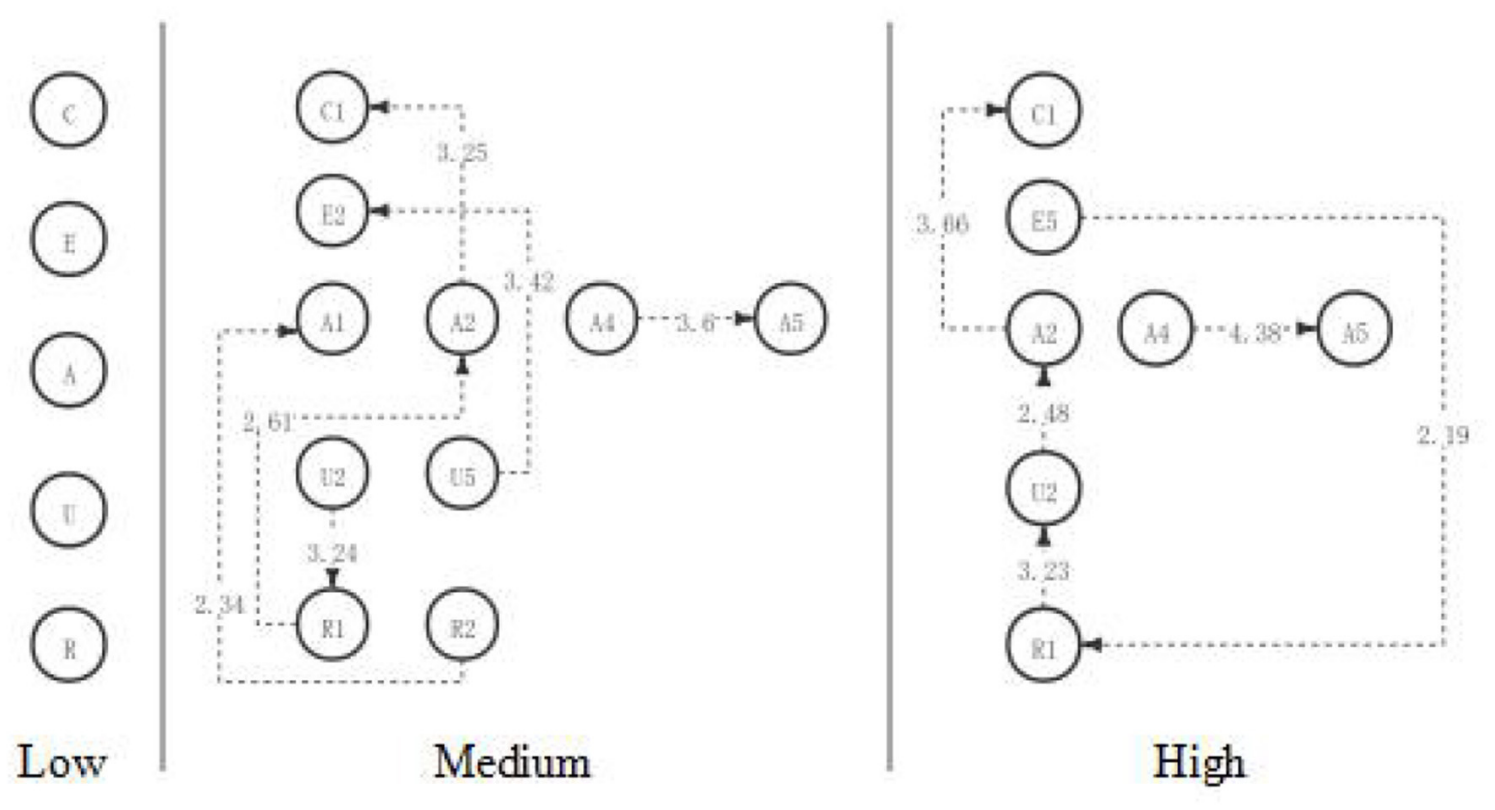
The critical thinking discussion activity path diagram of the different engagement group.

Based on the above results, in the second discussion, students with high, medium, and low academic engagement showed significant differences in the visualized critical thinking activity path diagrams in the discussion activities (as shown in Figure 7). The low engagement group did not produce a significant behavior sequence in this discussion, which is consistent with the hypothesis. What is inconsistent with the hypothesis is that although both the medium engagement group and the high engagement group have reached the highest level of critical thinking—creation, the significant behavior path of the medium engagement group is significantly better than that of the high engagement group.

### Critical Thinking Pathways of Participants With Different Levels of Academic Engagement After the Third STEAM Course

According to the coding results, the characteristics of critical thinking pathways of students in low, medium, and high academic engagement groups were analyzed. The specific results are analyzed as follows.

In the third discussion, there were two effective sequences, U2→A4 and C1→R1, in the low academic engagement group (Table 14). There is no sequence of behaviors with significant residuals between behaviors (Table 15).

**Table 14:** Behavioral adjustment residual values of critical thinking process in the low engagement group.

**Given:**	**R1**	**R2**	**U1**	**U2**	**U3**	**U4**	**U5**	**U6**	**A1**	**A2**	**A3**	**A4**	**A5**	**A6**	**E1**	**E2**	**E3**	**E4**	**E5**	**E6**	**C1**	**C2**	**C3**	**N1**	**N2**	**Totals**
R1	0	0	0	0	0	0	0	0	0	0	0	0	0	0	0	0	0	0	0	0	0	0	0	0	0	0
R2	0	0	0	0	0	0	0	0	0	0	0	0	0	0	0	0	0	0	0	0	0	0	0	0	0	0
U1	0	0	0	0	0	0	0	0	0	0	0	0	0	0	0	0	0	0	0	0	0	0	0	0	0	0
U2	0	0	0	0	0	0	0	0	0	0	0	1	0	0	0	0	0	0	0	0	0	0	0	0	0	1
U3	0	0	0	0	0	0	0	0	0	0	0	0	0	0	0	0	0	0	0	0	0	0	0	0	0	0
U4	0	0	0	0	0	0	0	0	0	0	0	0	0	0	0	0	0	0	0	0	0	0	0	0	0	0
U5	0	0	0	0	0	0	0	0	0	0	0	0	0	0	0	0	0	0	0	0	0	0	0	0	0	0
U6	0	0	0	0	0	0	0	0	0	0	0	0	0	0	0	0	0	0	0	0	0	0	0	0	0	0
A1	0	0	0	0	0	0	0	0	0	0	0	0	0	0	0	0	0	0	0	0	0	0	0	0	0	0
A2	0	0	0	0	0	0	0	0	0	0	0	0	0	0	0	0	0	0	0	0	0	0	0	0	0	0
A3	0	0	0	0	0	0	0	0	0	0	0	0	0	0	0	0	0	0	0	0	0	0	0	0	0	0
A4	0	0	0	0	0	0	0	0	0	0	0	0	0	0	0	0	0	0	0	0	0	0	0	0	0	0
A5	0	0	0	0	0	0	0	0	0	0	0	0	0	0	0	0	0	0	0	0	0	0	0	0	0	0
A6	0	0	0	0	0	0	0	0	0	0	0	0	0	0	0	0	0	0	0	0	0	0	0	0	0	0
E1	0	0	0	0	0	0	0	0	0	0	0	0	0	0	0	0	0	0	0	0	0	0	0	0	0	0
E2	0	0	0	0	0	0	0	0	0	0	0	0	0	0	0	0	0	0	0	0	0	0	0	0	0	0
E3	0	0	0	0	0	0	0	0	0	0	0	0	0	0	0	0	0	0	0	0	0	0	0	0	0	0
E4	0	0	0	0	0	0	0	0	0	0	0	0	0	0	0	0	0	0	0	0	0	0	0	0	0	0
E5	0	0	0	0	0	0	0	0	0	0	0	0	0	0	0	0	0	0	0	0	0	0	0	0	0	0
E6	0	0	0	0	0	0	0	0	0	0	0	0	0	0	0	0	0	0	0	0	0	0	0	0	0	0
C1	1	0	0	0	0	0	0	0	0	0	0	0	0	0	0	0	0	0	0	0	0	0	0	0	0	1
C2	0	0	0	0	0	0	0	0	0	0	0	0	0	0	0	0	0	0	0	0	0	0	0	0	0	0
C3	0	0	0	0	0	0	0	0	0	0	0	0	0	0	0	0	0	0	0	0	0	0	0	0	0	0
N1	0	0	0	0	0	0	0	0	0	0	0	0	0	0	0	0	0	0	0	0	0	0	0	0	0	0
N2	0	0	0	0	0	0	0	0	0	0	0	0	0	0	0	0	0	0	0	0	0	0	0	0	0	0
Totals	1	0	0	0	0	0	0	0	0	0	0	1	0	0	0	0	0	0	0	0	0	0	0	0	0	2

**Table 15:** Behavioral adjustment residual values of critical thinking process in the low engagement group.

**Given:**	**R1**	**R2**	**U1**	**U2**	**U3**	**U4**	**U5**	**U6**	**A1**	**A2**	**A3**	**A4**	**A5**	**A6**	**E1**	**E2**	**E3**	**E4**	**E5**	**E6**	**C1**	**C2**	**C3**	**N1**	**N2**
R1	0	0	0	0	0	0	0	0	0	0	0	0	0	0	0	0	0	0	0	0	0	0	0	0	0
R2	0	0	0	0	0	0	0	0	0	0	0	0	0	0	0	0	0	0	0	0	0	0	0	0	0
U1	0	0	0	0	0	0	0	0	0	0	0	0	0	0	0	0	0	0	0	0	0	0	0	0	0
U2	−1.41	0	0	0	0	0	0	0	0	0	0	1.41	0	0	0	0	0	0	0	0	0	0	0	0	0
U3	0	0	0	0	0	0	0	0	0	0	0	0	0	0	0	0	0	0	0	0	0	0	0	0	0
U4	0	0	0	0	0	0	0	0	0	0	0	0	0	0	0	0	0	0	0	0	0	0	0	0	0
U5	0	0	0	0	0	0	0	0	0	0	0	0	0	0	0	0	0	0	0	0	0	0	0	0	0
U6	0	0	0	0	0	0	0	0	0	0	0	0	0	0	0	0	0	0	0	0	0	0	0	0	0
A1	0	0	0	0	0	0	0	0	0	0	0	0	0	0	0	0	0	0	0	0	0	0	0	0	0
A2	0	0	0	0	0	0	0	0	0	0	0	0	0	0	0	0	0	0	0	0	0	0	0	0	0
A3	0	0	0	0	0	0	0	0	0	0	0	0	0	0	0	0	0	0	0	0	0	0	0	0	0
A4	0	0	0	0	0	0	0	0	0	0	0	0	0	0	0	0	0	0	0	0	0	0	0	0	0
A5	0	0	0	0	0	0	0	0	0	0	0	0	0	0	0	0	0	0	0	0	0	0	0	0	0
A6	0	0	0	0	0	0	0	0	0	0	0	0	0	0	0	0	0	0	0	0	0	0	0	0	0
E1	0	0	0	0	0	0	0	0	0	0	0	0	0	0	0	0	0	0	0	0	0	0	0	0	0
E2	0	0	0	0	0	0	0	0	0	0	0	0	0	0	0	0	0	0	0	0	0	0	0	0	0
E3	0	0	0	0	0	0	0	0	0	0	0	0	0	0	0	0	0	0	0	0	0	0	0	0	0
E4	0	0	0	0	0	0	0	0	0	0	0	0	0	0	0	0	0	0	0	0	0	0	0	0	0
E5	0	0	0	0	0	0	0	0	0	0	0	0	0	0	0	0	0	0	0	0	0	0	0	0	0
E6	0	0	0	0	0	0	0	0	0	0	0	0	0	0	0	0	0	0	0	0	0	0	0	0	0
C1	1.41	0	0	0	0	0	0	0	0	0	0	−1.41	0	0	0	0	0	0	0	0	0	0	0	0	0
C2	0	0	0	0	0	0	0	0	0	0	0	0	0	0	0	0	0	0	0	0	0	0	0	0	0
C3	0	0	0	0	0	0	0	0	0	0	0	0	0	0	0	0	0	0	0	0	0	0	0	0	0
N1	0	0	0	0	0	0	0	0	0	0	0	0	0	0	0	0	0	0	0	0	0	0	0	0	0
N2	0	0	0	0	0	0	0	0	0	0	0	0	0	0	0	0	0	0	0	0	0	0	0	0	0

In the third course, there were 12 kinds and 19 effective single sequences, such as U2→A4, R2→A4, and R2→A4, in the medium school engagement group, as shown in Table 16. The third discussion produced more sequences, mainly for understanding and seeking relevant content → analyzing and explaining viewpoints (U2→A4), with a total of four sequences, and relatively few sequences for the process of creating (C).

**Table 16:** Behavioral adjustment residual values of critical thinking process in the medium engagement group.

**Given:**	**R1**	**R2**	**U1**	**U2**	**U3**	**U4**	**U5**	**U6**	**A1**	**A2**	**A3**	**A4**	**A5**	**A6**	**E1**	**E2**	**E3**	**E4**	**E5**	**E6**	**C1**	**C2**	**C3**	**N1**	**N2**	**Totals**
R1	0	0	0	0	0	0	0	0	0	0	0	0	0	0	0	0	0	0	0	0	0	0	0	0	0	0
R2	0	0	0	0	0	0	0	0	1	1	0	2	0	0	0	2	0	0	0	0	0	0	0	0	0	6
U1	0	0	0	0	0	0	0	0	0	0	0	0	0	0	0	0	0	0	0	0	0	0	0	0	0	0
U2	2	1	0	0	0	0	0	0	0	1	0	4	0	0	0	0	0	0	0	0	0	0	0	0	0	8
U3	0	0	0	0	0	0	0	0	0	0	0	0	0	0	0	0	0	0	0	0	0	0	0	0	0	0
U4	0	0	0	0	0	0	0	0	0	0	0	0	0	0	0	0	0	0	0	0	0	0	0	0	0	0
U5	0	0	0	0	0	0	0	0	0	0	0	0	0	0	0	0	0	0	0	0	0	0	0	0	0	0
U6	0	0	0	0	0	0	0	0	0	0	0	0	0	0	0	0	0	0	0	0	0	0	0	0	0	0
A1	0	0	0	0	0	0	0	0	0	0	0	0	0	0	0	0	0	0	0	0	0	0	0	0	0	0
A2	1	0	0	0	0	0	0	0	0	0	0	0	0	0	0	0	0	0	0	0	2	0	0	0	0	3
A3	0	0	0	0	0	0	0	0	0	0	0	0	0	0	0	0	0	0	0	0	0	0	0	0	0	0
A4	0	0	0	0	0	0	0	0	0	0	0	0	0	0	0	0	0	0	0	0	0	0	0	0	0	0
A5	0	0	0	0	0	0	0	0	0	0	0	0	0	0	0	0	0	0	0	0	0	0	0	0	0	0
A6	0	0	0	0	0	0	0	0	0	0	0	0	0	0	0	0	0	0	0	0	0	0	0	0	0	0
E1	0	0	0	0	0	0	0	0	0	0	0	0	0	0	0	0	0	0	0	0	0	0	0	0	0	0
E2	0	0	0	0	0	0	0	0	0	0	0	0	0	0	0	0	1	0	0	0	0	0	0	0	0	1
E3	0	0	0	0	0	0	0	0	0	0	0	0	0	0	0	0	0	0	0	0	0	0	0	0	0	0
E4	0	0	0	0	0	0	0	0	0	0	0	0	0	0	0	0	0	0	0	0	0	0	0	0	0	0
E5	0	0	0	0	0	0	0	0	0	0	0	0	0	0	0	0	0	0	0	0	0	0	0	0	0	0
E6	0	0	0	0	0	0	0	0	0	0	0	0	0	0	0	0	0	0	0	0	0	0	0	0	0	0
C1	0	0	0	0	0	0	0	0	0	0	0	0	0	0	0	0	0	0	0	0	0	0	0	0	0	0
C2	0	0	0	0	0	0	0	0	0	0	0	0	0	0	0	0	0	0	0	0	0	0	0	0	0	0
C3	0	0	0	0	0	0	0	0	0	0	0	0	0	0	0	0	0	0	0	0	0	0	0	0	0	0
N1	1	0	0	0	0	0	0	0	0	0	0	0	0	0	0	0	0	0	0	0	0	0	0	0	0	1
N2	0	0	0	0	0	0	0	0	0	0	0	0	0	0	0	0	0	0	0	0	0	0	0	0	0	0
Totals	4	1	0	0	0	0	0	0	1	2	0	6	0	0	0	2	1	0	0	0	2	0	0	0	0	19

**Figure 8:**
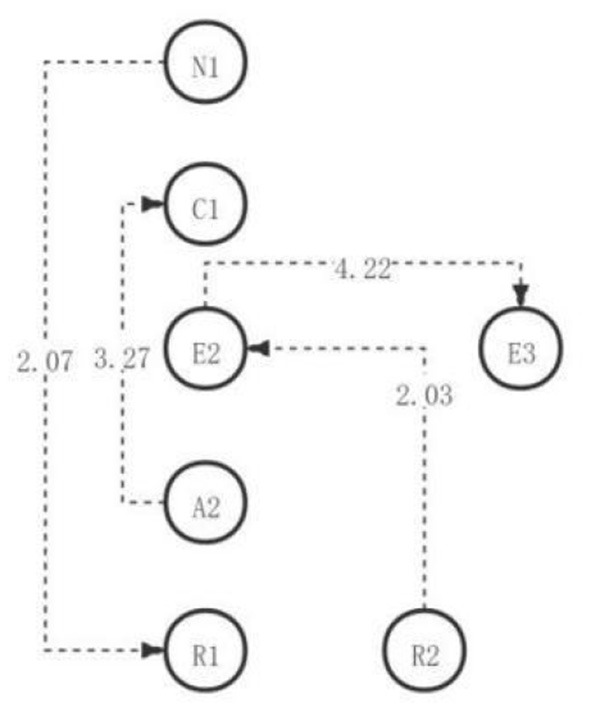
The critical thinking discussion activity path diagram of the medium engagement group.

Among them, there were four significant sequences with residual values >1.96 (Table 17), that is, identification approval problem → critical hypothesis (R2→E2), evidence classification → application implementation plan (A2→C1), critical hypothesis → detection consistency and inconsistency (E2→E3), and unrelated topic → identification restatement problem (N1→R1). After identifying the approval problem (as shown in Figure 8), medium school students in the academic engagement group would be making critical recognition of the current view (R2→E2). Based on classifying the collected evidence, students will directly implement the new ideas and plans (A2→C1). After criticizing the hypothesis, students will further test the consistency and inconsistency of the current opinion (E2→E3). After presenting some irrelevant content, some students will further clarify the problem to be solved, to refocus their thoughts on the topic (N1→R1).

**Table 17:** Behavioral adjustment residual values of critical thinking process in the medium engagement group.

**Given:**	**R1**	**R2**	**U1**	**U2**	**U3**	**U4**	**U5**	**U6**	**A1**	**A2**	**A3**	**A4**	**A5**	**A6**	**E1**	**E2**	**E3**	**E4**	**E5**	**E6**	**C1**	**C2**	**C3**	**N1**	**N2**
R1	0	0	0	0	0	0	0	0	0	0	0	0	0	0	0	0	0	0	0	0	0	0	0	0	0
R2	−1.55	0	0	0	0	0	0	0	1.48	0.32	0	0.06	0	0	0	2.03^*^	−0.71	0	0	0	−1.03	0	0	0	0
U1	0	0	0	0	0	0	0	0	0	0	0	0	0	0	0	0	0	0	0	0	0	0	0	0	0
U2	0.47	0.72	0	0	0	0	0	0	−0.85	0.05	0	1.64	0	0	0	−1.28	−0.85	0	0	0	−1.24	0	0	0	0
U3	0	0	0	0	0	0	0	0	0	0	0	0	0	0	0	0	0	0	0	0	0	0	0	0	0
U4	0	0	0	0	0	0	0	0	0	0	0	0	0	0	0	0	0	0	0	0	0	0	0	0	0
U5	0	0	0	0	0	0	0	0	0	0	0	0	0	0	0	0	0	0	0	0	0	0	0	0	0
U6	0	0	0	0	0	0	0	0	0	0	0	0	0	0	0	0	0	0	0	0	0	0	0	0	0
A1	0	0	0	0	0	0	0	0	0	0	0	0	0	0	0	0	0	0	0	0	0	0	0	0	0
A2	0.47	−0.56	0	0	0	0	0	0	−0.46	0	0	−1.33	0	0	0	−0.69	−0.46	0	0	0	3.27^*^	0	0	0	0
A3	0	0	0	0	0	0	0	0	0	0	0	0	0	0	0	0	0	0	0	0	0	0	0	0	0
A4	0	0	0	0	0	0	0	0	0	0	0	0	0	0	0	0	0	0	0	0	0	0	0	0	0
A5	0	0	0	0	0	0	0	0	0	0	0	0	0	0	0	0	0	0	0	0	0	0	0	0	0
A6	0	0	0	0	0	0	0	0	0	0	0	0	0	0	0	0	0	0	0	0	0	0	0	0	0
E1	0	0	0	0	0	0	0	0	0	0	0	0	0	0	0	0	0	0	0	0	0	0	0	0	0
E2	−0.55	−0.3	0	0	0	0	0	0	−0.25	−0.4	0	−0.72	0	0	0	0	4.22^*^	0	0	0	−0.36	0	0	0	0
E3	0	0	0	0	0	0	0	0	0	0	0	0	0	0	0	0	0	0	0	0	0	0	0	0	0
E4	0	0	0	0	0	0	0	0	0	0	0	0	0	0	0	0	0	0	0	0	0	0	0	0	0
E5	0	0	0	0	0	0	0	0	0	0	0	0	0	0	0	0	0	0	0	0	0	0	0	0	0
E6	0	0	0	0	0	0	0	0	0	0	0	0	0	0	0	0	0	0	0	0	0	0	0	0	0
C1	0	0	0	0	0	0	0	0	0	0	0	0	0	0	0	0	0	0	0	0	0	0	0	0	0
C2	0	0	0	0	0	0	0	0	0	0	0	0	0	0	0	0	0	0	0	0	0	0	0	0	0
C3	0	0	0	0	0	0	0	0	0	0	0	0	0	0	0	0	0	0	0	0	0	0	0	0	0
N1	2.07^*^	−0.29	0	0	0	0	0	0	−0.24	−0.38	0	−0.68	0	0	0	−0.35	−0.24	0	0	0	−0.34	0	0	0	0
N2	0	0	0	0	0	0	0	0	0	0	0	0	0	0	0	0	0	0	0	0	0	0	0	0	0

* *Indicates that the Z-score value is significant*.

In the third discussion, there were seven kinds and 12 effective single sequences, such as U2→R1 and U2→A2, R2→E5, in the medium and high school academic engagement group, as shown in Table 18. In the third discussion, more single sequences were generated, mainly identifying the approval question → supporting argument (R2→E5). There were four sequences in total.

**Table 18:** Behavioral adjustment residual values of critical thinking process in the high engagement group.

**Given:**	**R1**	**R2**	**U1**	**U2**	**U3**	**U4**	**U5**	**U6**	**A1**	**A2**	**A3**	**A4**	**A5**	**A6**	**E1**	**E2**	**E3**	**E4**	**E5**	**E6**	**C1**	**C2**	**C3**	**N1**	**N2**	**Totals**
R1	0	0	0	0	0	0	0	0	0	0	0	0	0	0	0	0	0	0	0	0	0	0	0	0	0	0
R2	1	0	0	0	0	0	1	0	0	0	0	2	0	0	0	0	0	0	3	0	0	0	0	0	0	7
U1	0	0	0	0	0	0	0	0	0	0	0	0	0	0	0	0	0	0	0	0	0	0	0	0	0	0
U2	2	0	0	0	0	0	0	0	0	2	0	0	0	0	0	0	0	0	0	0	0	0	0	0	0	4
U3	0	0	0	0	0	0	0	0	0	0	0	0	0	0	0	0	0	0	0	0	0	0	0	0	0	0
U4	0	0	0	0	0	0	0	0	0	0	0	0	0	0	0	0	0	0	0	0	0	0	0	0	0	0
U5	0	0	0	0	0	0	0	0	0	0	1	0	0	0	0	0	0	0	0	0	0	0	0	0	0	1
U6	0	0	0	0	0	0	0	0	0	0	0	0	0	0	0	0	0	0	0	0	0	0	0	0	0	0
A1	0	0	0	0	0	0	0	0	0	0	0	0	0	0	0	0	0	0	0	0	0	0	0	0	0	0
A2	0	0	0	0	0	0	0	0	0	0	0	0	0	0	0	0	0	0	0	0	0	0	0	0	0	0
A3	0	0	0	0	0	0	0	0	0	0	0	0	0	0	0	0	0	0	0	0	0	0	0	0	0	0
A4	0	0	0	0	0	0	0	0	0	0	0	0	0	0	0	0	0	0	0	0	0	0	0	0	0	0
A5	0	0	0	0	0	0	0	0	0	0	0	0	0	0	0	0	0	0	0	0	0	0	0	0	0	0
A6	0	0	0	0	0	0	0	0	0	0	0	0	0	0	0	0	0	0	0	0	0	0	0	0	0	0
E1	0	0	0	0	0	0	0	0	0	0	0	0	0	0	0	0	0	0	0	0	0	0	0	0	0	0
E2	0	0	0	0	0	0	0	0	0	0	0	0	0	0	0	0	0	0	0	0	0	0	0	0	0	0
E3	0	0	0	0	0	0	0	0	0	0	0	0	0	0	0	0	0	0	0	0	0	0	0	0	0	0
E4	0	0	0	0	0	0	0	0	0	0	0	0	0	0	0	0	0	0	0	0	0	0	0	0	0	0
E5	0	0	0	0	0	0	0	0	0	0	0	0	0	0	0	0	0	0	0	0	0	0	0	0	0	0
E6	0	0	0	0	0	0	0	0	0	0	0	0	0	0	0	0	0	0	0	0	0	0	0	0	0	0
C1	0	0	0	0	0	0	0	0	0	0	0	0	0	0	0	0	0	0	0	0	0	0	0	0	0	0
C2	0	0	0	0	0	0	0	0	0	0	0	0	0	0	0	0	0	0	0	0	0	0	0	0	0	0
C3	0	0	0	0	0	0	0	0	0	0	0	0	0	0	0	0	0	0	0	0	0	0	0	0	0	0
N1	0	0	0	0	0	0	0	0	0	0	0	0	0	0	0	0	0	0	0	0	0	0	0	0	0	0
N2	0	0	0	0	0	0	0	0	0	0	0	0	0	0	0	0	0	0	0	0	0	0	0	0	0	0
Totals	3	0	0	0	0	0	1	0	0	2	1	2	0	0	0	0	0	0	3	0	0	0	0	0	0	12

**Figure 9:**
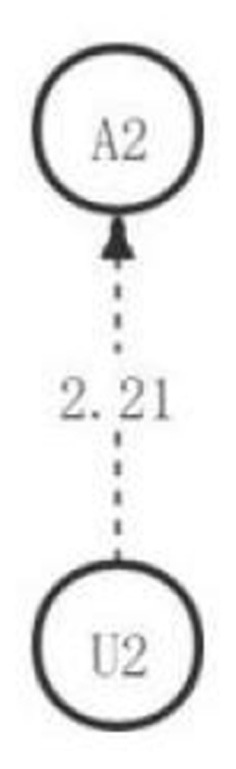
The critical thinking discussion activity path diagram of the high engagement group.

Among them, there is one significant sequence with a residual value >1.96 (as shown in Table 19), that is, understanding and searching for relevant content → evidence classification and classification (U2→A2). As shown in Figure 9, students in the medium and high engagement group in the third discussion would further categorize or classify the current evidence (U2→A2) based on understanding the problem and searching for relevant content.

**Table 19:** Behavioral adjustment residual values of critical thinking process in the high engagement group.

**Given:**	**R1**	**R2**	**U1**	**U2**	**U3**	**U4**	**U5**	**U6**	**A1**	**A2**	**A3**	**A4**	**A5**	**A6**	**E1**	**E2**	**E3**	**E4**	**E5**	**E6**	**C1**	**C2**	**C3**	**N1**	**N2**
R1	0	0	0	0	0	0	0	0	0	0	0	0	0	0	0	0	0	0	0	0	0	0	0	0	0
R2	−1	0	0	0	0	0	0.74	0	0	−1.83	−1.23	1.33	0	0	0	0	0	0	1.72	0	0	0	0	0	0
U1	0	0	0	0	0	0	0	0	0	0	0	0	0	0	0	0	0	0	0	0	0	0	0	0	0
U2	1.43	0	0	0	0	0	−0.77	0	0	2.21^*^	−0.74	−1.09	0	0	0	0	0	0	−1.41	0	0	0	0	0	0
U3	0	0	0	0	0	0	0	0	0	0	0	0	0	0	0	0	0	0	0	0	0	0	0	0	0
U4	0	0	0	0	0	0	0	0	0	0	0	0	0	0	0	0	0	0	0	0	0	0	0	0	0
U5	−0.63	0	0	0	0	0	0	0	0	−0.49	3.29^*^	−0.49	0	0	0	0	0	0	−0.63	0	0	0	0	0	0
U6	0	0	0	0	0	0	0	0	0	0	0	0	0	0	0	0	0	0	0	0	0	0	0	0	0
A1	0	0	0	0	0	0	0	0	0	0	0	0	0	0	0	0	0	0	0	0	0	0	0	0	0
A2	0	0	0	0	0	0	0	0	0	0	0	0	0	0	0	0	0	0	0	0	0	0	0	0	0
A3	0	0	0	0	0	0	0	0	0	0	0	0	0	0	0	0	0	0	0	0	0	0	0	0	0
A4	0	0	0	0	0	0	0	0	0	0	0	0	0	0	0	0	0	0	0	0	0	0	0	0	0
A5	0	0	0	0	0	0	0	0	0	0	0	0	0	0	0	0	0	0	0	0	0	0	0	0	0
A6	0	0	0	0	0	0	0	0	0	0	0	0	0	0	0	0	0	0	0	0	0	0	0	0	0
E1	0	0	0	0	0	0	0	0	0	0	0	0	0	0	0	0	0	0	0	0	0	0	0	0	0
E2	0	0	0	0	0	0	0	0	0	0	0	0	0	0	0	0	0	0	0	0	0	0	0	0	0
E3	0	0	0	0	0	0	0	0	0	0	0	0	0	0	0	0	0	0	0	0	0	0	0	0	0
E4	0	0	0	0	0	0	0	0	0	0	0	0	0	0	0	0	0	0	0	0	0	0	0	0	0
E5	0	0	0	0	0	0	0	0	0	0	0	0	0	0	0	0	0	0	0	0	0	0	0	0	0
E6	0	0	0	0	0	0	0	0	0	0	0	0	0	0	0	0	0	0	0	0	0	0	0	0	0
C1	0	0	0	0	0	0	0	0	0	0	0	0	0	0	0	0	0	0	0	0	0	0	0	0	0
C2	0	0	0	0	0	0	0	0	0	0	0	0	0	0	0	0	0	0	0	0	0	0	0	0	0
C3	0	0	0	0	0	0	0	0	0	0	0	0	0	0	0	0	0	0	0	0	0	0	0	0	0
N1	0	0	0	0	0	0	0	0	0	0	0	0	0	0	0	0	0	0	0	0	0	0	0	0	0
N2	0	0	0	0	0	0	0	0	0	0	0	0	0	0	0	0	0	0	0	0	0	0	0	0	0

* *Indicates that the Z-score value is significant*.

**Figure 10:**
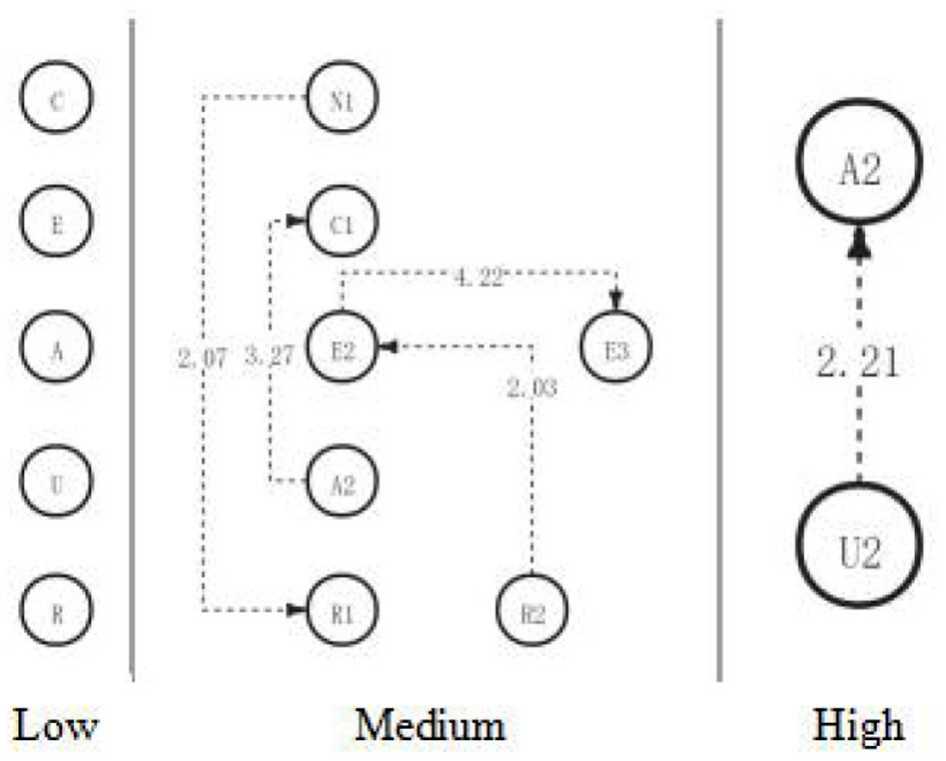
The critical thinking discussion activity path diagram of the different engagement group.

Based on the above results, in the third discussion, students with high, medium, and low academic engagement showed significant differences in their critical thinking activity path diagrams in the discussion activities (as shown in Figure 10). Consistent with the hypothesis, the low engagement group had the worst significant behavior path of critical thinking among the three groups and did not produce a significant behavior sequence. Contrary to the hypothesis, the medium engagement group had a significantly better sequence of behaviors than the high engagement group and reached the highest level of critical thinking—creativity. The high engagement group only reached the analytical level.

### Comparison of Critical Thinking Levels of Participants With Different Academic Engagement Levels in Three STEAM Courses

**Figure 11:**
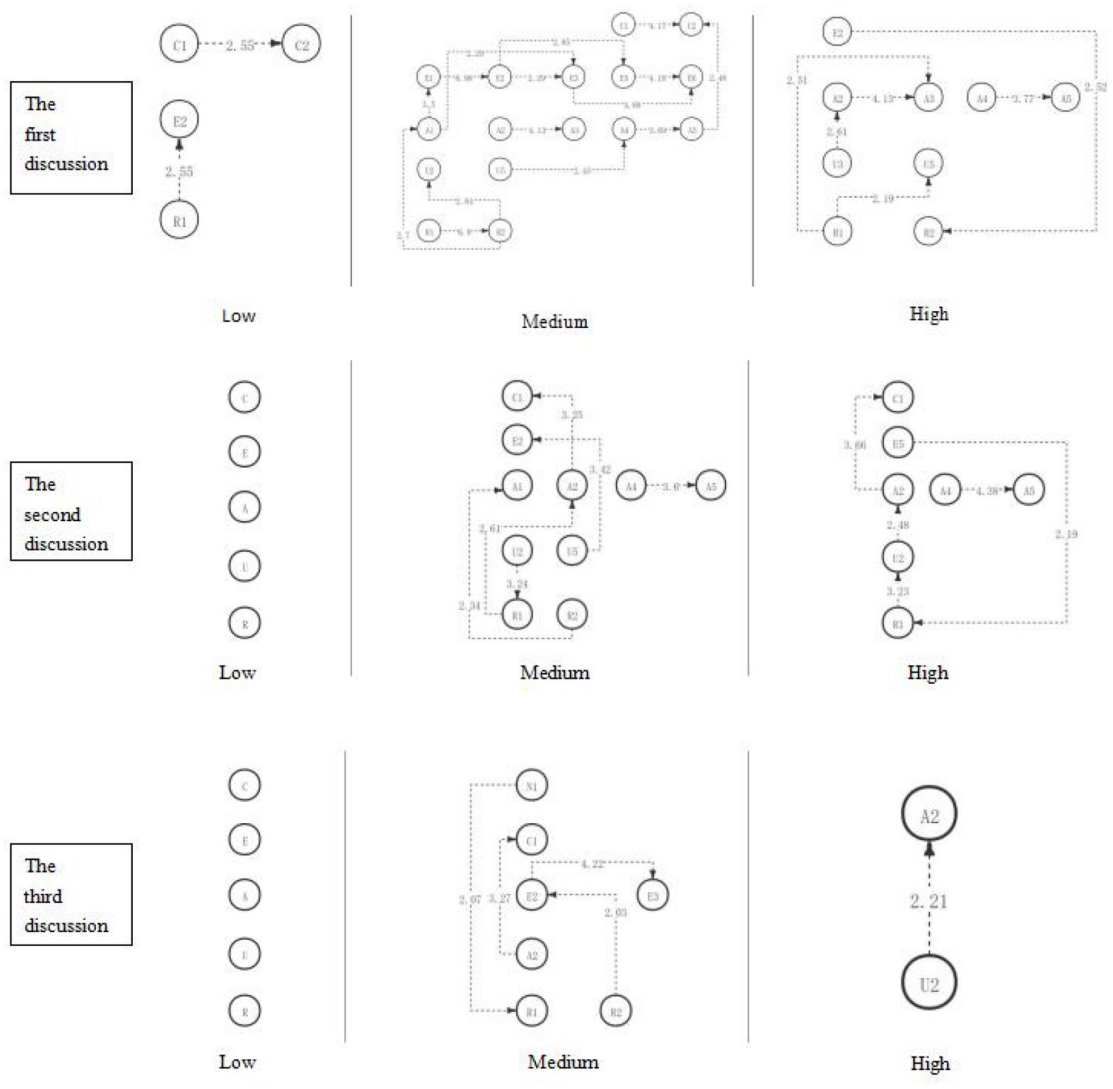
The critical thinking discussion activity path diagram of the different engagement group.

With the longitudinal comparison of the three-course discussions, it is found that students in the low engagement group show a low level of critical thinking behavior sequence in the three discussions (as shown in Figure 11). With the implementation of the STEAM course, there is no influence on the critical thinking behavior sequence of students. Under the implementation of the STEAM course, all the students in the engagement group showed more significant behavior sequences of critical thinking, and their critical thinking level reached the highest level—creation; students in the high engagement group also showed significant critical thinking behavior sequence, but their critical thinking level did not reach the level of creativity.

## Discussion

This study examined the differences in critical thinking among students with different levels of academic engagement in STEAM courses. The results show that there are significant differences in critical thinking levels among students with different levels of academic engagement in STEAM courses. Moreover, students with medium academic engagement have the highest level of critical thinking in the STEAM course, which is superior to those in the high academic engagement group, while students with low academic engagement have the lowest level of critical thinking. Although the researchers have done some studies on the relationship among STEAM education methods, academic engagement, and critical thinking level, this study found the moderating effect of academic engagement on the development of critical thinking level in STEAM courses.

Different levels of academic engagement also reflect different characteristics in the five levels of critical thinking from low to high. Among the students having a low engagement, there were few effective sequences and significant behavior sequences in the discussions of three STEAM courses. In the few significant behavior sequences, the level of critical thinking reached the level of creation, but such a situation did not continue to appear in the three discussions, and no effective single sequence or significant behavior sequence appeared in the two discussions. This may be due to a variety of reasons, such as students in the low academic engagement group did not actively participate in the discussion, did not fully understand the task topic, or did not focus on the discussion task. The students with a medium level of academic engagement showed good critical thinking levels in the three discussions, and there were more effective behavior sequences and significant behavior sequences in the three discussions. However, the effective behavior sequence and significant behavior sequence of the high academic engagement group were less than those of the medium academic engagement group. This seems to be different from the views mentioned in the existing studies that students with high engagement are energetic and not tired in learning activities, have a high degree of study concentration, and can actively participate in class discussions (Fredricks et al., 2004). The reasons for this situation need further research to determine the influence of other factors on the critical thinking level of students.

This research has made some theoretical contributions to the cultivation of critical thinking of students by a STEAM education method. First, compared with previous studies, this study considered the moderating effect of academic engagement on the STEAM curriculum and critical thinking level. Future research needs to explore the synergistic effects of academic interest of students (Pan, 2017), learning motivation (Liang and Lu, 2019), etc. Second, the measurement of the critical thinking level of students in this study is based on the content analysis of the online discussion texts in the STEAM course. Compared with the previous measurement method of self-report, this study makes full use of the process data to dig deeply into the changes in the critical thinking level of students in the learning process.

The study also provides some practical guidance for promoting critical thinking in STEAM education: first, to guide students to participate more actively in course discussions, such as designing thematic tasks to attract students; second, timely guidance should be provided to students with low academic engagement to improve their learning participation.

## Open Data

The data has not yet loaded online. However, we will send the data by request. The request should be sent to Jingrong Sha: shajr818@163.com.

## Data Availability Statement

The raw data supporting the conclusions of this article will be made available by the authors, without undue reservation.

## Ethics Statement

This research was approved by the Ethics Committee of the College of Educational Science and Technology at Northwest Minzu University. Participants were volunteers who provided written informed consent.

## Author Contributions

JS designed and implemented the experiments. HS is responsible for collecting data and analyzed the data. All the authors participated in the writing of the article and approved the submitted version.

## Conflict of Interest

The authors declare that the research was conducted in the absence of any commercial or financial relationships that could be construed as a potential conflict of interest.

## Publisher's Note

All claims expressed in this article are solely those of the authors and do not necessarily represent those of their affiliated organizations, or those of the publisher, the editors and the reviewers. Any product that may be evaluated in this article, or claim that may be made by its manufacturer, is not guaranteed or endorsed by the publisher.
